# Supplementary material for: Entanglement of dark electron-nuclear spin defects in diamond
Source: Nat Commun. 2021 Jun 9;12:3470. doi: 10.1038/s41467-021-23454-9 (PMC8190113; doi:10.1038/s41467-021-23454-9)
Supplement: Supplementary file 1 — Supplementary Information [file 41467_2021_23454_MOESM1_ESM.pdf]

Supplementary Information for "Entanglement of dark electron-nuclear spin defects in diamond"

CONTENTS

1. SUPPLEMENTARY NOTE: System Hamiltonian 2
A. P1 center 2
B. NV-P1 system 2
C. Orientation in the crystal lattice 3

2. SUPPLEMENTARY NOTE: DEER and DEER(y) sequence 3
A. DEER 3
B. DEER(y) 4
C. Multiple P1 centers 4

3. SUPPLEMENTARY NOTE: JT dependent coupling 5

4. SUPPLEMENTARY NOTE: Fitting the Hamiltonian parameters 5

5. SUPPLEMENTARY NOTE: Estimate of P1 concentration 8

6. SUPPLEMENTARY NOTE: Relaxation times of S1 during repetitive DEER measurements 10

7. SUPPLEMENTARY NOTE: Preparation of P1 bath configurations by active optical reset 10
A. Optimization to increase experimental rate 10
B. Active optical reset 12

8. SUPPLEMENTARY NOTE: Correlation measurements for different JT axes 13

9. SUPPLEMENTARY NOTE: NV-P1 dipolar coupling sign in $|+1, \mathrm{A}\rangle$ 15

10. SUPPLEMENTARY NOTE: NV-P1 coupling in $|+1, \mathrm{A}\rangle$ 16

11. SUPPLEMENTARY NOTE: Magnetic field stability 17

12. SUPPLEMENTARY NOTE: Coherence times of S3/S4 in $|+1, \mathrm{D}\rangle$ 18

13. SUPPLEMENTARY NOTE: Effective gyromagnetic ratio and spin coherence 18

14. SUPPLEMENTARY NOTE: Entanglement sequence 19

15. SUPPLEMENTARY NOTE: Optimization of initialisation/readout 20
A. ^{14}N and JT state 20
B. Electron spin initialisation and readout 20
C. Optimization of sequential initialisation in $|+1, \mathrm{D}\rangle$ and $|+1, \mathrm{A}\rangle$ 21

16. SUPPLEMENTARY NOTE: NV fluorescence rate reference 22

References 22

1. SUPPLEMENTARY NOTE: SYSTEM HAMILTONIAN

In this note we describe the Hamiltonian of a system consisting of a P1 center and an NV center in diamond.

A. P1 center

The Hamiltonian of the P1 center is given by [1]:

$$H_{\mathrm{P1_i}} = \gamma_e \mathbf{B}\cdot\mathbf{S} + \mathbf{S}\cdot\hat{\mathbf{A}}_{\mathbf{i}}\cdot\mathbf{I} + \gamma_n \mathbf{B}\cdot\mathbf{I} + \mathbf{I}\cdot\hat{\mathbf{P}}_{\mathbf{i}}\cdot\mathbf{I}, \tag{1}$$

where γ_e ($\approx 2\pi \cdot 2.802$ MHz/G) and γ_n ($\approx -2\pi \cdot 0.3078$ kHz/G) are the electron and ^{14}N gyromagnetic ratio respectively. $\mathbf{B}$ is the external magnetic field vector and $\mathbf{S}$ and $\mathbf{I}$ are the electron spin-1/2 and nuclear spin-1 operator vectors. The P1 center exhibits a JT distortion along the axis of one of the four carbon-nitrogen bonds (denoted by $i \in \{\mathrm{A,B,C,D}\}$). This JT axis defines the principal axis of the hyperfine interaction and the quadrupole interaction.

The hyperfine tensor ($\hat{\mathbf{A}}_{\mathbf{i}}$) and quadrupole tensor ($\hat{\mathbf{P}}_{\mathbf{i}}$) can be obtained in any coordinate frame via a transformation of the diagonal hyperfine tensor, $\mathbf{A_{diag}} = \mathrm{diag}[A_x, A_y, A_z]$ and diagonal quadrupole tensor, $\mathbf{P_{diag}} = \mathrm{diag}[P_x, P_y, P_z]$:

$$\hat{\mathbf{A}}_{\mathbf{i}} = R^T \cdot \mathbf{A_{diag}} \cdot R \tag{2}$$

and

$$\hat{\mathbf{P}}_{\mathbf{i}} = R^T \cdot \mathbf{P_{diag}} \cdot R. \tag{3}$$

Here R is the rotation matrix from the principal axis of the P1 center to any other coordinate frame defined by Euler angles $\{\alpha, \beta, \gamma\}$:

$$R(\alpha,\beta,\gamma) = \begin{pmatrix} \cos(\gamma)\cos(\beta)\cos(\alpha) - \sin(\gamma)\sin(\alpha) & \cos(\gamma)\cos(\beta)\sin(\alpha) + \sin(\gamma)\cos(\alpha) & -\cos(\gamma)\sin(\beta) \\ -\sin(\gamma)\cos(\beta)\cos(\alpha) - \cos(\gamma)\sin(\alpha) & -\sin(\gamma)\cos(\beta)\sin(\alpha) + \cos(\gamma)\cos(\alpha) & \sin(\gamma)\sin(\beta) \\ \sin(\beta)\cos(\alpha) & \sin(\beta)\sin(\alpha) & \cos(\beta) \end{pmatrix}. \tag{4}$$

Due to the axial symmetry of the P1 center in its principal axis coordinate frame ($A_x = A_y$ and $P_x = P_y$), this can be reduced to:

$$R(\beta,\alpha) = \begin{pmatrix} \cos(\beta)\cos(\alpha) & \cos(\beta)\sin(\alpha) & -\sin(\beta) \\ -\sin(\alpha) & \cos(\alpha) & 0 \\ \sin(\beta)\cos(\alpha) & \sin(\beta)\sin(\alpha) & \cos(\beta) \end{pmatrix}. \tag{5}$$

For any of the four JT axes, the hyperfine (quadrupole) tensor $\hat{\mathbf{A}}_{\mathbf{i}}$ ($\hat{\mathbf{P}}_{\mathbf{i}}$) in the coordinate frame of the symmetry axis of the NV center is obtained via a transformation with angles $(\beta,\alpha)_i$, where $(\beta,\alpha)_i \in \{(109.5^\circ, 240^\circ)_\mathrm{C}, (109.5^\circ, 120^\circ)_\mathrm{B}, (109.5^\circ, 0^\circ)_\mathrm{A}, (0^\circ, 0^\circ)_\mathrm{D}\}$.

B. NV-P1 system

The Hamiltonian of a coupled NV-P1 system (in the frame of the symmetry axis of the NV center) is given by the terms corresponding to the NV, the P1 and the terms describing their dipolar coupling:

$$H_{\mathrm{tot}} = H_{\mathrm{NV}} + H_{\mathrm{P1}} + H_{\mathrm{dipole}}. \tag{6}$$

For the NV we only consider the electron spin of the NV center:

$$H_{\mathrm{NV}} = \Delta J_z^2 + \gamma_e \mathbf{B}\cdot\mathbf{J}. \tag{7}$$

where $\Delta = 2\,\pi \cdot 2.877$ GHz is the zero-field splitting and $\mathbf{J}$ is the electron spin 1 vector.
If we consider a point dipole coupling between the NV and the electron spin of the P1 center, separated by a vector $\mathbf{r}$, the dipole term can be written as:

$$H_{\mathrm{dipole}} = \nu_{\mathrm{dip}} \cdot \big(3(\mathbf{J}\cdot\hat{\mathbf{r}})(\mathbf{S}\cdot\hat{\mathbf{r}}) - \mathbf{J}\cdot\mathbf{S}\big), \tag{8}$$

where $\nu_{\text{dip}} = \frac{-\mu_0 \gamma_e \gamma_e \hbar}{4\pi r^3}$, $r = |\mathbf{r}|$ and $\hat{\mathbf{r}} = \mathbf{r}/r$. Transforming to spherical coordinates using the definitions $r_x = r\sin(\theta)\cos(\varphi)$, $r_y = r\sin(\theta)\sin(\varphi)$ and $r_z = r\cos(\theta)$, gives

$$\begin{aligned} H_{\text{dipole}} = \nu_{\text{dip}} \cdot \Big[& J_x S_x \left(3\sin^2(\theta)\cos^2(\varphi) - 1\right) + J_y S_y \left(3\sin^2(\theta)\sin^2(\varphi) - 1\right) + J_z S_z \left(3\cos^2(\theta) - 1\right) \\ & + (J_y S_x + J_x S_y) 3\sin^2(\theta)\cos(\varphi)\sin(\varphi) + (J_z S_x + J_x S_z) 3\cos(\theta)\sin(\theta)\cos(\varphi) \\ & + (J_z S_y + J_y S_z) 3\cos(\theta)\sin(\theta)\sin(\varphi) \Big]. \end{aligned} \tag{9}$$

Due to the large difference between electron and ^{14}N gyromagnetic ratio ($\gamma_e/\gamma_n \sim 9000$) we expect the electron-nuclear NV-P1 dipolar coupling to be negligible and omit this term in the Hamiltonian.

C. Orientation in the crystal lattice

In the diamond lattice, the P1 defect is located in a tetrahedral geometry with four surrounding carbon atoms. A single carbon atom can be positioned either directly above (orientation 1) or below (orientation 2) the P1 center's nitrogen atom, and the other three carbon atoms at 109.5° bond angles below or above respectively.

If we consider the JT distortions along the **z** axis (JT axis D) for these two orientations, the nitrogen atom either distorts in the **-z** direction or the +**z** direction. Therefore, these two orientations correspond to two spin Hamiltonians as in Supplementary Eq. (6): $H_{\text{tot},1}$ with $\hat{\mathbf{A}}_{\mathbf{D}} = \mathbf{A}_{\mathbf{diag}}$ and $H_{\text{tot},2}$ with $\hat{\mathbf{A}}_{\mathbf{D}} = R(180°, 0°)^T \cdot \mathbf{A}_{\mathbf{diag}} \cdot R(180°, 0°)$ (similar for $\hat{\mathbf{P}}_{\mathbf{D}}$). From Supplementary Eq. (5) it is evident that $R(180°, 0°)^T \cdot \mathbf{A}_{\mathbf{diag}} \cdot R(180°, 0°) = \mathbf{A}_{\mathbf{diag}}$, and thus $H_{\text{tot},1} = H_{\text{tot},2}$. Therefore, in the experiments performed in this work, we cannot distinguish between these two different orientations of P1 centers.

2. SUPPLEMENTARY NOTE: DEER AND DEER(Y) SEQUENCE

Here a more detailed description is given for the experimental sequences shown in Figs. 2b and 3b of the main text. We consider an idealized case of a single NV, a single P1 center and their magnetic dipolar coupling. In a diagonalized frame, the energy eigenstates and eigenvalues of the system are labelled as $|m_s, m_{\uparrow/\downarrow}, m_I\rangle$ and $\lambda_{m_s, m_{\uparrow/\downarrow}, m_I}$ respectively. The eigenstates form a 12-dimensional Hilbert space (the subspace m$_s$ = {0,-1} of the NV, the electron spin 1/2 of the P1 center and its ^{14}N spin 1).

A. DEER

We consider the sequence of unitary operations as applied during a DEER sequence with a single repetition K=1 (see Fig. 2b in the main text). First we apply $U_{\text{NV},1} = R_x(\pi/2)_{\text{NV}} \otimes \mathbb{1}_{\text{P1}}$, a $\pi/2$ rotation on the NV with phase x, followed by an evolution time $U_{\text{evo}}(\tau)$, which is given by diag$[e^{-i\lambda_{0,\uparrow,+1}\cdot\tau}$, $e^{-i\lambda_{0,\uparrow,0}\cdot\tau}$, ... ,$e^{-i\lambda_{m_s,m_{\uparrow/\downarrow},m_I}\cdot\tau}$, ... ,$e^{-i\lambda_{-1,\downarrow,-1}\cdot\tau}]$. This is followed by a π pulse on the NV; $U_{\text{NV},2} = R_x(\pi)_{\text{NV}} \otimes \mathbb{1}_{\text{P1}}$. Simultaneously, we apply a π pulse on the P1 electron spin conditional on its ^{14}N state $|m_I\rangle = |+1\rangle$. This operation is described by $U_{\text{P1}} = \mathbb{1}_{\text{NV}} \otimes R_x(\pi)_{\text{P1,e}} \otimes |+1\rangle\langle +1| + \mathbb{1}_{\text{NV}} \otimes \mathbb{1}_{\text{P1,e}} \otimes |0\rangle\langle 0| + \mathbb{1}_{\text{NV}} \otimes \mathbb{1}_{\text{P1,e}} \otimes |-1\rangle\langle -1|$. Then there is another evolution time $U_{\text{evo}}(\tau)$ and finally a $\pi/2$ pulse $U_{\text{NV},3} = R_{-x}(\pi/2)_{\text{NV}} \otimes \mathbb{1}_{\text{P1}}$ with phase $-x$. We assume perfect π and $\pi/2$ pulses between energy eigenstates. For an initial state ρ_1, we obtain a final state given by:

$$\rho_{f,1}(\tau) = U_{\text{NV},3} U_{\text{evo}}(\tau) U_{\text{P1}} U_{\text{NV},2} U_{\text{evo}}(\tau) U_{\text{NV},1} \rho_1 U_{\text{NV},1}^\dagger U_{\text{evo}}(\tau)^\dagger U_{\text{NV},2}^\dagger U_{\text{P1}}^\dagger U_{\text{evo}}(\tau)^\dagger U_{\text{NV},3}^\dagger \tag{10}$$

For the NV initialized in $|0\rangle$ but mixed P1 electron and ^{14}N spin states, the initial density matrix is given as:

$$\rho_1 = \frac{1}{6} |0\rangle\langle 0| \otimes \mathbb{1}_{\text{P1}} \tag{11}$$

The reduced density matrix of the NV as a function of interaction time is given by:

$$\rho_{\text{NV}}(\tau) = \sum_{m_{\uparrow,\downarrow}, m_I} \langle m_{\uparrow,\downarrow}, m_I| \rho_{f,1}(\tau) |m_{\uparrow,\downarrow}, m_I\rangle = \frac{1}{6}\cos(\nu\cdot\tau)\big[\,|-1\rangle\langle -1| - |0\rangle\langle 0|\,\big] + \frac{1}{6}|0\rangle\langle 0| + \frac{5}{6}|-1\rangle\langle -1|. \tag{12}$$

Here the effective NV-P1 dipolar coupling (ν) is given as

$$\nu = \lambda_{-1,\downarrow,+1} - \lambda_{0,\downarrow,+1} - (\lambda_{-1,\uparrow,+1} - \lambda_{0,\uparrow,+1}). \tag{13}$$

Note that at $\tau = \pi/|\nu|$ we obtain the highest probability of measuring $|0\rangle_{\mathrm{NV}}$. Upon measurement of $|0\rangle_{\mathrm{NV}}$ we obtain the state:

$$\rho_{m_s=0} = \frac{|0\rangle\langle 0|\,\rho_{f,1}(\tau)\,|0\rangle\langle 0|}{\mathrm{Tr}(|0\rangle\langle 0|\,\rho_{f,1}(\tau))} = \frac{1}{2}\,|0\rangle\langle 0| \otimes \mathbb{1}_{\mathrm{P1,e}} \otimes |{+1}\rangle\langle{+1}|\,, \tag{14}$$

and thus initialize the ^{14}N state of the P1 center in $|{+1}\rangle$.

B. DEER(y)

Now we consider DEER(y) (see main text Fig. 3b), which is sensitive to the P1 electron spin state. This sequence is the DEER sequence with the phase of the final $\pi/2$ pulse changed from $-x \to -y$, or $-x \to +y$. For an initial state ρ_2 and the phase of the final $\pi/2$ pulse as $-y$, we obtain the final state $\rho_{f,2}$ by:

$$\rho_{f,2}(\tau) = U_{\mathrm{NV},4} U_{\mathrm{evo}}(\tau) U_{\mathrm{P1}} U_{\mathrm{NV},2} U_{\mathrm{evo}}(\tau) U_{\mathrm{NV},1} \rho_2 U^{\dagger}_{\mathrm{NV},1} U_{\mathrm{evo}}(\tau)^{\dagger} U^{\dagger}_{\mathrm{NV},2} U^{\dagger}_{\mathrm{P1}} U_{\mathrm{evo}}(\tau)^{\dagger} U^{\dagger}_{\mathrm{NV},4}. \tag{15}$$

Note that the final operator has been changed to $U_{\mathrm{NV},4} = R_{-y}(\pi/2)_{\mathrm{NV}} \otimes \mathbb{1}_{\mathrm{P1}}$ with phase $-y$. If we consider the NV initialized in $|0\rangle$ and the ^{14}N state of the P1 in $|{+1}\rangle$, the initial density matrix is given as:

$$\rho_2 = \frac{1}{2}\,|0\rangle\langle 0| \otimes \mathbb{1}_{\mathrm{P1,e}} \otimes |{+1}\rangle\langle{+1}|\,. \tag{16}$$

The reduced density matrix of the NV as a function of interaction time is now given by:

$$\rho_{\mathrm{NV}}(\tau) = \sum_{m_{\uparrow,\downarrow}, m_I} \langle m_{\uparrow,\downarrow}, m_I|\,\rho_{f,2}(\tau)\,|m_{\uparrow,\downarrow}, m_I\rangle = \frac{1}{2}\cos(\nu\cdot t)\big[\,|0\rangle\langle -1| + |{-1}\rangle\langle 0|\,\big] + \frac{1}{2}\,|0\rangle\langle 0| + \frac{1}{2}\,|{-1}\rangle\langle -1|\,. \tag{17}$$

Upon measurement of $|0\rangle_{\mathrm{NV}}$ we obtain the state:

$$\rho_{m_s=0} = \frac{|0\rangle\langle 0|\,\rho_{f,2}(\tau)\,|0\rangle\langle 0|}{\mathrm{Tr}(|0\rangle\langle 0|\,\rho_{f,2}(\tau))} = \frac{1}{2}(1+\sin(\nu\cdot\tau))\,|0,\uparrow,+1\rangle\langle 0,\uparrow,+1| + \frac{1}{2}(1-\sin(\nu\cdot\tau))\,|0,\downarrow,+1\rangle\langle 0,\downarrow,+1| \tag{18}$$

Note that at interaction time $\tau = \pi/(2|\nu|)$, the initialized state is either $|0,\downarrow,+1\rangle$ or $|0,\uparrow,+1\rangle$ depending on the sign of ν. Applying a phase $+y$ instead of $-y$ in the final $\pi/2$ pulse effectively changes the signs in front of the $\sin(\nu\cdot\tau)$ terms in Supplementary Eq. (18), which is used in Fig. 4b of the main text.

C. Multiple P1 centers

In this section we discuss multiple coupled P1 centers that simultaneously occupy the same nitrogen nuclear spin- and JT-state. For simplicity we discuss the case of two P1 centers - S1 and S2 - with significant couplings to the NV center, but this can be extended to more P1 centers. Given the measured coupling strengths in $|{+1},\mathrm{D}\rangle$ and the chosen interaction time 2τ as shown in Fig. 2c of the main text, the NV acquires a phase of approximately $\pm\pi$ ($\pm 4\pi/5$) due to S1 (S2) individually. For the case that both spins occupy the same $|m_I, i\rangle$ state, summation of coupling strengths and the number of repetitive measurements play a role for the observed effective phase. If both spins are in $|{+1},\mathrm{D}\rangle$, the total phase is given by the sum or difference of the individual phase contributions and depends on the parity of their electron spins. This results in phases of $\pm 1\pi/5$ or $\pm 9\pi/5$. However, because the P1 electron spin states relax rapidly under repeated measurement (see Supplementary Fig. 6b), for $K = 820$ the measured result is an average of the results generated by the phases of each possible two-spin state. In this case, this gives an effective phase of $1\pi/5$. The resulting signal is well separated from the cases of just S1 or S2, so that cases in which both P1s occupy the same state are removed by the initialization procedures used. Note also that the probability that two of the P1s (S1-S4) are in the same state simultaneously is small.

3. SUPPLEMENTARY NOTE: JT DEPENDENT COUPLING

Mainly due to the anisotropic hyperfine interaction of the P1 center, the effective NV-P1 dipolar coupling ν, which is the interaction observed in a DEER experiment (Supplementary Eq. (13)), varies depending on the JT axis. We numerically calculate ν as a function of angles θ and ϕ of the vector $\mathbf{r}$ between an NV and a P1 using an exemplary case. Supplementary Fig. 1a, shows the calculated $\nu/2\pi$ for different states $|m_I, i\rangle$ as a function of angle ϕ as in Supplementary Eq. (9) (here $\theta = 45°$). This figure demonstrates that the JT state can affect the effective dipolar coupling strength ν (Supplementary Eq. (13)). For a P1 center in the states $|+1,\mathrm{A}\rangle$,$|+1,\mathrm{B}\rangle$ and $|+1,\mathrm{C}\rangle$ the principal axis of the hyperfine tensor is rotated 109.5° w.r.t. the $\mathbf{z}$ axis (NV axis) and thus varying ϕ has a substantial effect on ν. For a P1 center in $|+1,\mathrm{D}\rangle$ the hyperfine tensor's principal axis is along $\mathbf{z}$ and therefore the dependence on ϕ is small. The observed dependence in the simulation for $|+1,\mathrm{D}\rangle$ is explained by the purposely slightly tilted magnetic field. The tilted magnetic field also explains the differences in maximal and minimal values of ν for the states $|+1,\mathrm{A}\rangle$,$|+1,\mathrm{B}\rangle$ and $|+1,\mathrm{C}\rangle$. The ϕ angles at which ν is at a maximum for these three states are shifted by $\Delta\phi \approx 120°$ w.r.t. each other as is expected from the three-fold rotational symmetry.

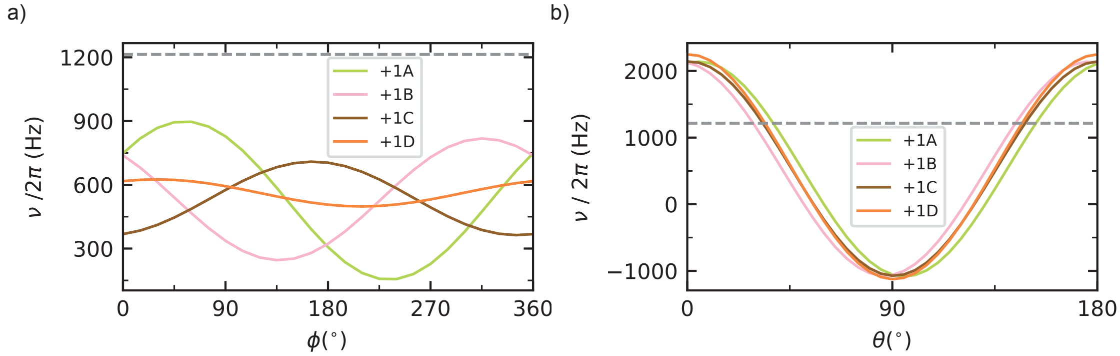

Supplementary Figure 1: Example showing JT dependent effective dipolar coupling ν. a) Numerical simulation of $\nu/2\pi$ as a function of angle ϕ of the vector $\mathbf{r}$ between the NV and a P1. As an example we set $\theta = 45°$ and $|\mathbf{r}| = 35$ nm. The magnetic field $\mathbf{B}$ and hyperfine/quadrupole parameters as obtained from the fitted DEER spectrum (main text Fig. 1b) are used. Colored curves indicate the P1 center in different states $|+1, i\rangle$. The dashed grey line indicates the value of $\nu_{\mathrm{dip}}/2\pi$ as in Supplementary Eq. (9). **b)** Similar to (a) but now as a function of θ ($\phi = 90°$).

Supplementary Fig. 1b illustrates that the relative differences of ν for different states are largest near the magic angles $\theta = \pm 54.7°$. Note that information about the P1 positions can be obtained by combining the knowledge of which signal belongs to which P1 center for several $|m_I, i\rangle$ states (Fig. 2d main text) and measuring the NV-P1 dipolar coupling. This provides a future opportunity to determine the position of P1 centers (up to inversion symmetry) w.r.t. the NV.

4. SUPPLEMENTARY NOTE: FITTING THE HAMILTONIAN PARAMETERS

This section describes the fitting procedure used to obtain the Hamiltonian parameters $A_\parallel$, $A_\perp$, P, B_x, B_y and B_z from the DEER spectroscopy (see Fig. 1b and Supplementary Fig. 2). We use parabolic fits of the measured dips to determine their center frequencies, and subsequently use a least squares method to minimize the difference between the measured frequencies and the frequencies resulting from diagonalization of the Hamiltonian (Supplementary Eq. (1)) for all 4 JT axes (see Supplementary Fig. 2b). This method requires that we can assign which measured frequency (dips in Supplementary Fig. 2a) belongs to which transition. We use a two step process. First, we take the four highest-energy transitions, which are well separated from any other transition, and use initial values for $A_\parallel$, $A_\perp$, P [2] to obtain an estimate of the magnetic field vector. Second, we select 11 well-isolated transitions and corresponding experimental dips to fit $A_\parallel$, $A_\perp$, P and the magnetic field vector.

To obtain an initial estimate of B_x, B_y and B_z, we first perform a brute force optimization. We sweep B_x and B_y between ±4 G and B_z between 45 and 46 G in discrete steps and diagonalize the Hamiltonian (Supplementary Eq. (1)), where we use $\gamma_e = 2\pi \cdot$ 2.802495 MHz/G and γ_n = - $2\pi \cdot$ 0.3078 kHz/G. For every combination we sort the four highest frequencies (corresponding to $|+1, i\rangle$) and calculate $\Delta = \sum_{i=1}^{4}(f_{i,\mathrm{exp}} - f_{i,\mathrm{theo}})^2$, where $f_{i,\mathrm{theo}}$ is the

transition frequency obtained by diagonalization and $f_{i,\mathrm{exp}}$ is the measured frequency. We find a minimum in Δ for $\mathbf{B} = (2.53, 1.39, 45.37)^T$ G.

We then use 11 dips that we can unambiguously assign to a well-isolated transition and measure these dips with high accuracy, see Supplementary Fig. 2b. We use this data to extract $A_\parallel$, $A_\perp$, P and the magnetic field vector using a least squares fit. The fit obtains $\{A_\parallel, A_\perp, P_\parallel\}$ = {114.0264(9), 81.312(1), -3.9770(9)} MHz and $\mathbf{B}$ = { 2.437(2), 1.703(1), 45.5553(5)} G. The vertical lines in Supplementary Fig. 2b are the 11 transition frequencies calculated with these values. In order to provide a more quantitative comparison we provide the experimentally measured frequencies of the 11 dips in Supplementary Table 1 alongside with their values calculated from Supplementary Eq. (1). Furthermore, by inspection, we identify 9 other dips in Supplementary Fig. 2a. We determine their center frequency and compare them to their closest transition frequency resulting from the fitted parameters, see Supplementary Table 1.

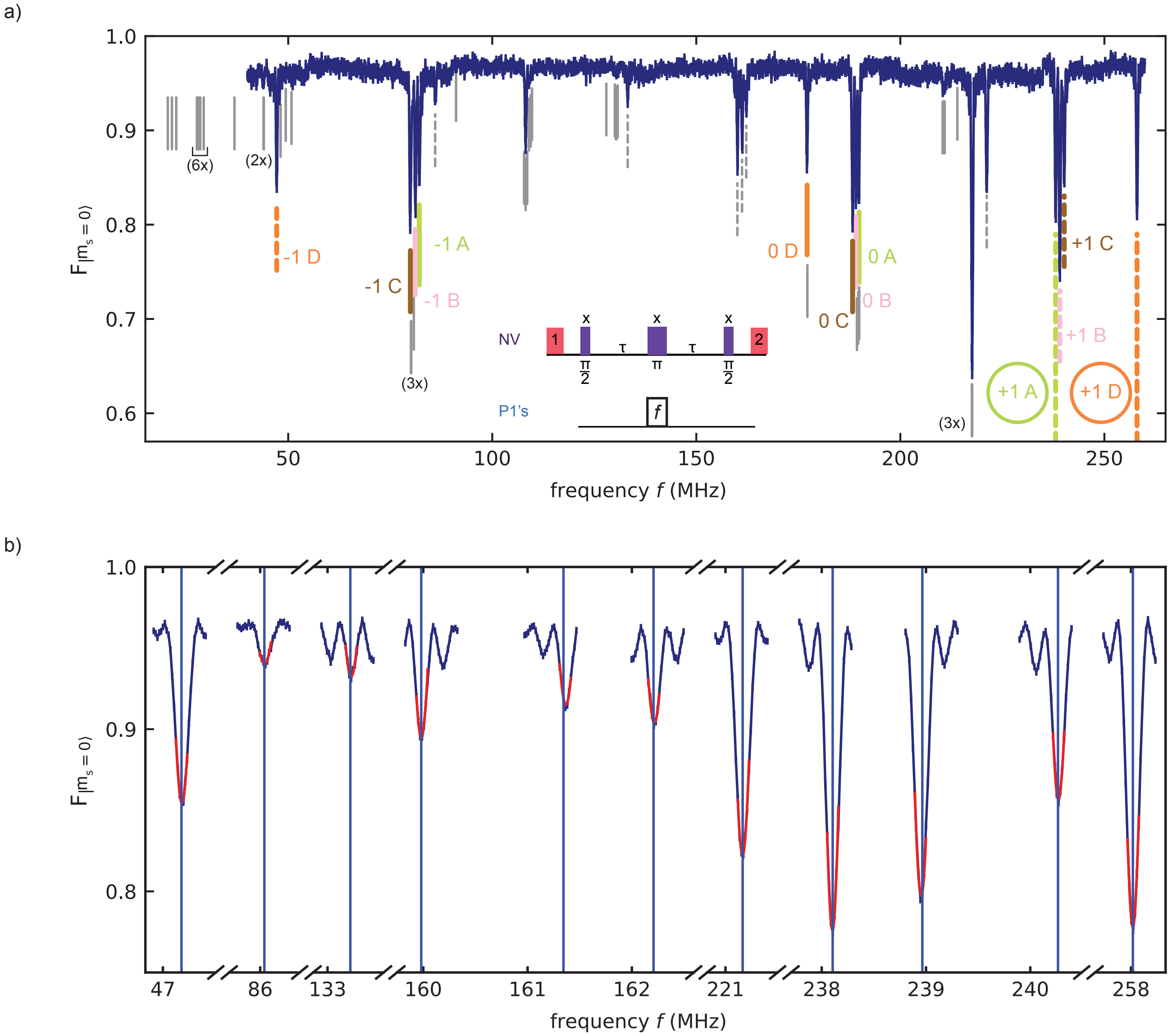

Supplementary Figure 2: Fitting the P1 Hamiltonian parameters. a) Complete dataset corresponding to Fig. 1b (main text). No measurements were performed around the predicted transitions on the left. b) Measurement of 11 dips that are assigned to a well isolated transition (blue lines). Side lobes are due to Rabi oscillations. We fit a parabola (red) and extract the center frequency. Using a least squares fit of the center frequencies and the transitions, we extract the values of $\{A_\parallel, A_\perp, P_\parallel\}$ and $\mathbf{B}$. Error bars indicate one statistical standard deviation, with a typical value 2×10^{-3}, which is smaller than the data points.

Dip Nr	Experimental value (MHz)	Supplementary Eq. (1) (MHz)	$\|m_I, i\rangle$ state
1	47.183(1)	47.179	$\|-1,\mathrm{D}\rangle$
2	86.042(2)	86.040	
3	133.227(2)	133.219	
4	159.9810(4)	159.980	
5	161.367(4)	161.344	
6	162.217(3)	162.208	
7	221.1641(7)	221.1648	
8	238.1027(6)	238.1051	$\|+1,\mathrm{A}\rangle$
9	238.954(1)	238.965	$\|+1,\mathrm{B}\rangle$
10	240.271(1)	240.266	$\|+1,\mathrm{C}\rangle$
11	258.0176(6)	258.018	$\|+1,\mathrm{D}\rangle$
12	79.898(4)	79.961	$\|-1,\mathrm{C}\rangle$
13	81.16(2)	81.10	$\|-1,\mathrm{B}\rangle$
14	82.106(8)	82.119	$\|-1,\mathrm{A}\rangle$
15	108.18(2)	108.20	
16	177.1167(2)	177.1476	$\|0,\mathrm{D}\rangle$
17	188.32(2)	188.32	$\|0,\mathrm{C}\rangle$
18	189.15(6)	189.11	$\|0,\mathrm{B}\rangle$
19	189.88(5)	189.88	$\|0,\mathrm{A}\rangle$
20	217.5783(8)	217.5709	

Supplementary Table 1: Comparison of measured P1 transition frequencies (Supplementary Fig. 2b) with closest values from Supplementary Eq. (1). We include the 11 frequencies of Supplementary Fig. 2b used in the fitting, as well as 9 other identified dips in Supplementary Fig. 2a, which is taken at a slightly different magnetic field compared to Supplementary Fig. 2b.

5. SUPPLEMENTARY NOTE: ESTIMATE OF P1 CONCENTRATION

In this section we estimate the P1 concentration surrounding the NV center. We calculate an expected concentration (C_d) based on decoherence of the NV electron spin. In the secular approximation the dipolar interaction of a number (N_d) of P1 centers and the NV (see Supplementary Eq. (9)) is given as:

$$H_{\text{int}} = J_z \sum_{k=1}^{N_d} 2\pi \cdot \nu_k \cdot (1 - 3\cos^2(\theta)) \cdot S_z = J_z \sum_{k=1}^{N_d} b_k \cdot S_z, \tag{19}$$

where $2\pi \cdot \nu_k = \frac{-\mu_0 \gamma_e \gamma_e \hbar}{4\pi r_k^3}$ and θ the polar angle in spherical coordinates. For a given concentration of P1 centers C_d, we consider a sphere around the NV center of radius R:

$$R = \left(\frac{3 \cdot V_{\text{tot}}}{4\pi} \right)^{1/3}, \tag{20}$$

where $V_{\text{tot}} = \frac{N_d \cdot V_{\text{unit}}}{C_d \cdot 8}$ with V_{unit} the volume of a diamond crystal unit cell, and 8 denotes the number of atoms within one unit cell. Within such a sphere we generate a number of P1 centers ($N_d = 40$) at random positions and calculate T_2^* due to a Gaussian spin bath [3] as:

$$T_2^* = \frac{\sqrt{2}}{\frac{1}{2}\sqrt{\sum_{k=1}^{N_d} b_k^2}}. \tag{21}$$

We repeat this procedure to generate $m = 10^4$ different spatial configurations of P1 centers and calculate the average, $\langle T_2^* \rangle$.

The result of this simulation as a function of P1 concentration C_d is shown in Supplementary Fig. 3. For randomly positioned P1s, $1/\langle T_2^* \rangle$ scales linearly with C_d. Note the difference in scaling with the average number of P1 centers coupled to during a DEER measurement, $\langle n_{\text{spins}} \rangle$, which scales as $1/T_{2,\text{DEER}} \propto \sqrt{\langle n_{\text{spins}} \rangle}$ due to the fixed positions of the P1s [4]. We estimate the NV decoherence time due to coupling to P1s to be within two values (as indicated by the red vertical lines in Supplementary Fig. 3). The first value is the measured $T_{2,\text{NV}}^* = 97(3)$ μs ($1/T_{2,\text{NV}}^* \approx 10$ kHz). Here it is assumed that the P1 centers are the dominant spin bath and the effect of the ^{13}C bath, and other magnetic field noise sources, on the NV decoherence is small. The second value is extrapolated from the measured $T_{2,\text{DEER}} = 0.803(5)$ ms for $|+1, \text{D}\rangle$ and thus is given by $T_2^* \sim T_{2,\text{DEER}}/\sqrt{12} \approx 230$ μs ($1/T_2^* \approx 4$ kHz). Here we assume approximately equal coupling strength in all 12 $|m_I, i\rangle$ states and an equal probability of occurrence for each state. The concentration is expected to be within these values and thus we estimate $C_d \sim 75$ ppb.

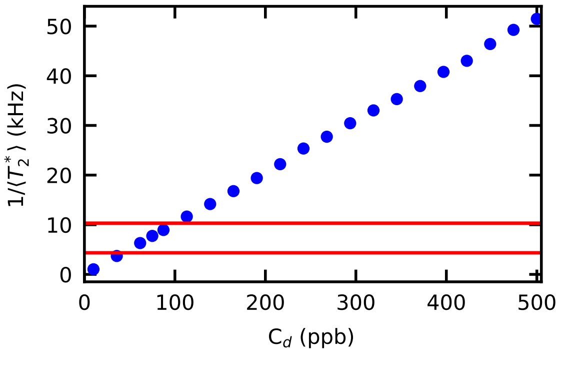

Supplementary Figure 3: **Simulated $1/\langle T_2^* \rangle$.** Simulation based on m = 10^4 different defect configurations of $N_d = 40$ defects. The red vertical lines indicate estimated bounds for $1/\langle T_2^* \rangle$.

Supplementary Fig. 4 shows the probability density of $b_k/2\pi$ for the first four P1 spins, given a concentration C_d = 75 ppb and the condition 4 kHz $\leq 1/T_2^* \leq$ 10 kHz for the NV spin. These distributions demonstrate the expected values of $|b_k|$ to be close to the measured values as stated in Methods (indicated by the vertical red lines). The probability densities without a condition on $1/T_2^*$ are shown in Supplementary Fig. 5. These plots show that given the concentration $C_d \sim 75$ ppb, a large range of coupling strengths are possible for the nearest spin (including the measured value |17.8(5)| kHz as measured between S1 and S2).

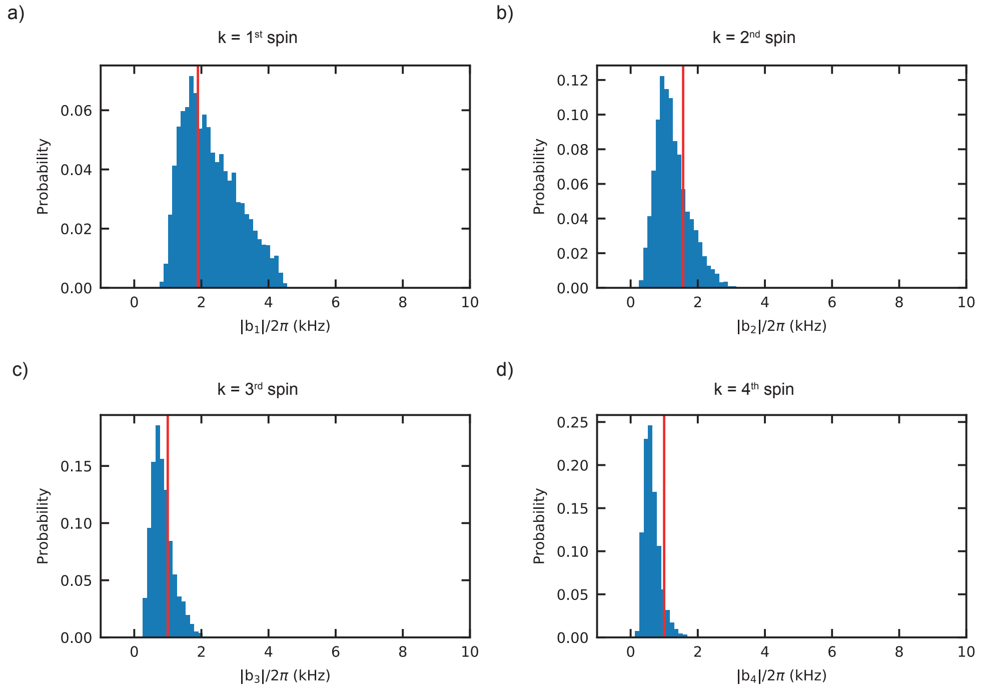

Supplementary Figure 4: **Simulated distributions of the coupling strengths for the most strongly coupled P1 spins.** a),b),c),d) Distributions of $|b_k|/2\pi$ given 4 kHz $\leq 1/T_2^* \leq$ 10 kHz and a concentration C_d = 75 ppb.

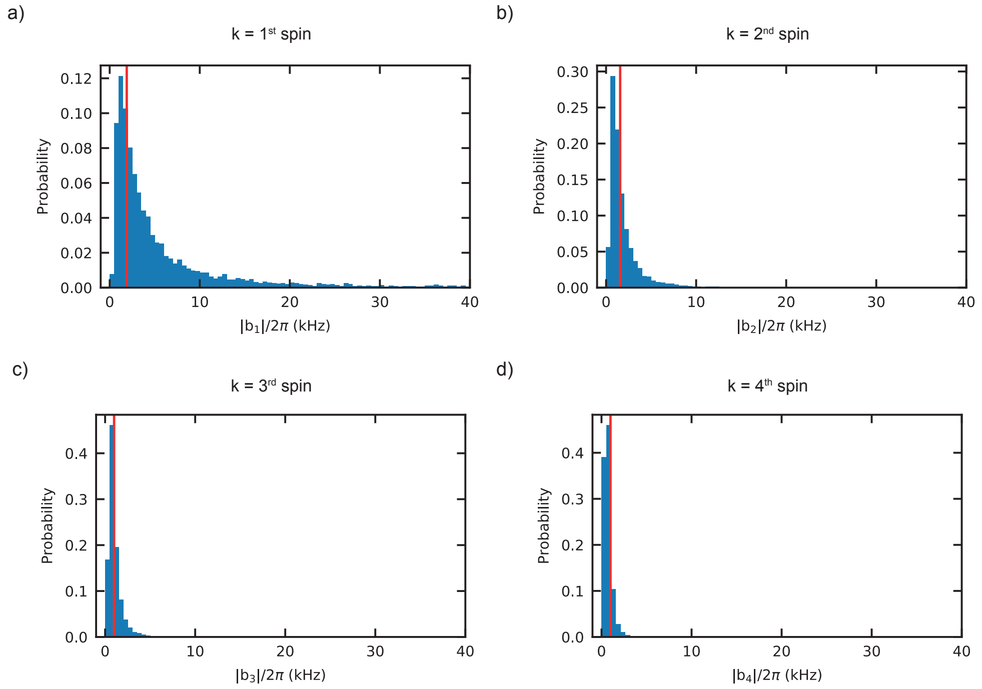

Supplementary Figure 5: **Simulated distributions for the 4 most strongly coupled spins.** a),b),c),d) Distributions of $|b_k|/2\pi$ given a concentration C_d = 75 ppb.

6. SUPPLEMENTARY NOTE: RELAXATION TIMES OF S1 DURING REPETITIVE DEER MEASUREMENTS

This section characterizes the relaxation of the P1 $|+1,\mathrm{D}\rangle$ state (Supplementary Fig. 6a) and the P1 electron spin in $|+1,\mathrm{D}\rangle$ (Supplementary Fig. 6b) under repeated measurement sequences. We first prepare spin S1 in $|+1,\mathrm{D}\rangle$ using DEER measurements (K=820) and subsequently apply 5 sequential sets of DEER measurements with K=820. We plot the result of each set in Supplementary Fig. 6a. We obtain a 1/e decay of ∼19 sets, showing the $|+1,\mathrm{D}\rangle$ state is stable over $\sim 1.5\times 10^4$ DEER repetitions (including optical 637 nm pulses).

To investigate the stability of the P1 electron state under repetitive DEER(y) measurements, we initialize S1 in $|+1,\mathrm{D}\rangle$ and prepare its electron state in $|\uparrow\rangle$ using L=8 DEER(y) measurements. Subsequently we apply 51 sequential sets of DEER(y) measurements with L=8 and plot the result of each set in Supplementary Fig. 6b. We extract a 1/e decay for $|\uparrow\rangle$ of ∼32 sets, showing a stability over ∼250 DEER(y) repetitions. Comparing this with the 1/e decay of $|+1,\mathrm{D}\rangle$ shows that a single sequence is much more destructive for the P1 electron state than for the combined nitrogen nuclear spin and JT state. This limits the amount of DEER(y) sequences L that can be used to initialize the P1 electron spin with high fidelity.

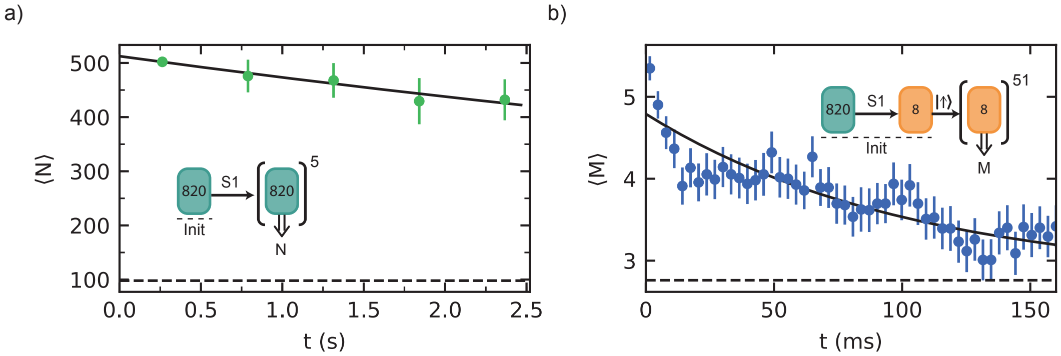

Supplementary Figure 6: Relaxation during repetitive readout. a) Relaxation from the $|+1,\mathrm{D}\rangle$ state while continuously performing DEER measurements. We fit (solid line) the curve to $o + A_0 \cdot e^{-t/T_{|+1,\mathrm{D}\rangle\mathrm{rep}}}$, where o is fixed to the uninitialized mean value (dashed line) and obtain $T_{|+1,\mathrm{D}\rangle\mathrm{rep}} = 10(4)$ s. This timescale corresponds to ∼ 19 bins of DEER measurements with K=820 repetitions. b) Relaxation from the $|\uparrow\rangle$ state, we fit (solid line) the curve to $o_1 + A_1 \cdot e^{-t/T_{|\uparrow\rangle\mathrm{rep}}}$, where o_1 is fixed to the uninitialized mean value (dashed line) and obtain $T_{|\uparrow\rangle\mathrm{rep}} = 103(8)$ ms. This timescale corresponds to ∼ 32 bins of DEER(y) measurements with L=8 repetitions.

7. SUPPLEMENTARY NOTE: PREPARATION OF P1 BATH CONFIGURATIONS BY ACTIVE OPTICAL RESET

Several of the experiments require initialisation of the charge, nitrogen nuclear spin and JT degrees of freedom for single P1 centers. To be able to distinguish the signals of the different P1 centers a large amount of measurement repetitions K is required, which limits the experimental repetition rate. In this section we describe how we increase the experimental rate fourfold by dynamically resetting the P1 center states based on outcomes of early measurement repetitions. The key ideas are: (1) cases for which none of the P1 centers start in the desired state can be identified already after a few repetitions, so that the sequence can be aborted, and (2) the state of the P1 centers can be rapidly reset by a laser pulse (515 nm) before re-attempting the initialization.

A. Optimization to increase experimental rate

In various experiments, we initialize the nitrogen nuclear spin- and JT-state of selected P1 centers by using the outcome of K = 420 or K = 820 DEER measurements to herald the desired state (Fig. 2a of the main text and Supplementary Fig. 9). In Supplementary Fig. 7a, we plot the histogram of $\sim 3\times 10^5$ DEER measurements in bins of K=420 on $|+1,\mathrm{D}\rangle$. In this dataset we define a successful (unsuccessful) initialisation of either S1 or S2 in $|+1,\mathrm{D}\rangle$ if the outcome N of K=420 DEER measurements fulfils $N > 180$ ($N \le 180$), see the green (red) part of the distribution in Supplementary Fig. 7a. We find ∼11% of the distribution in the green region which indicates the probability to find either S1 or S2 in $|+1,\mathrm{D}\rangle$. This differs from a probability of $\sim 2\cdot\frac{1}{12}$ and suggests that P1 centers occasionally ionize or preferentially occupy specific states. Research on all 12 nitrogen nuclear spin- and JT-states is required to

provide more insight on the occupation of such states and provides a potential direction for future research. We note that from Supplementary Fig. 9 we extract ∼11% of S1 or S2 being in $|+1, \mathrm{A}\rangle$. Because of these low probabilities, the success rate of initialising S1 or S2 is limited. In Supplementary Fig. 7b, we inspect the measurement outcome N1 of successful (green) and unsuccessful (red) initialisation attempts after the first K=Θ DEER sequences, where in this figure $\Theta = 5$. This shows that N1 is low for many unsuccessful cases (red), indicating that the experiment can be sped up by aborting the sequence after a few repetitions if N1 is below a threshold.

We implement a Monte Carlo method to determine a good set of parameters to increase the experimental rate. A schematic of the method is depicted in Supplementary Fig. 7d. We sample from the dataset in Supplementary Fig. 7a and check after Θ DEER measurements (DEER 1) whether the outcome N1 is below a threshold Λ. If so, we abort, sample again from the dataset and inspect DEER 1 again. If N1 $\geq \Lambda$, we continue until finishing 420 DEER measurements. If the outcome of the 420 DEER measurements is above 180, we accept it as a successful initialisation. We continuously sample from the dataset until we achieve 1000 successful runs (N1 + N2 > 180).

We now sweep Θ and Λ and calculate the average time $\langle T_{\mathrm{avg}} \rangle$ required to finish one successful run, where we average over 1000 successful runs (see Supplementary Fig. 7d). Here we use that 1 DEER sequence takes 684 µs and we introduce an overhead time of 1 ms for resetting the P1 center states (experimentally done with a laser pulse, see subsection below). Two critical parameters that determine $\langle T_{\mathrm{avg}} \rangle$ are 1) the probability P(N1 $\geq \Lambda$) to pass the condition in DEER 1 and 2) the probability P(N1 + N2 > 180|N1 $\geq \Lambda$) to obtain N1 + N2 > 180 conditioned on passing the condition in DEER 1. These probabilities can be extracted from Supplementary Fig. 7a and b and are shown in Supplementary Fig. 7c for the example case of $\Theta = 5$. From Supplementary Fig. 7d we extract that $\Theta = 5$ and $\Lambda = 3$ are parameters where $\langle T_{\mathrm{avg}} \rangle$ is small and we find an increase in experimental rate by a factor ∼ 5. This shows that the experiment can be sped up by aborting the sequence after 5 repetitions already if N1 is below 3. Note, however, that this method assumes perfect randomization of the P1 center states by drawing random samples from the dataset.

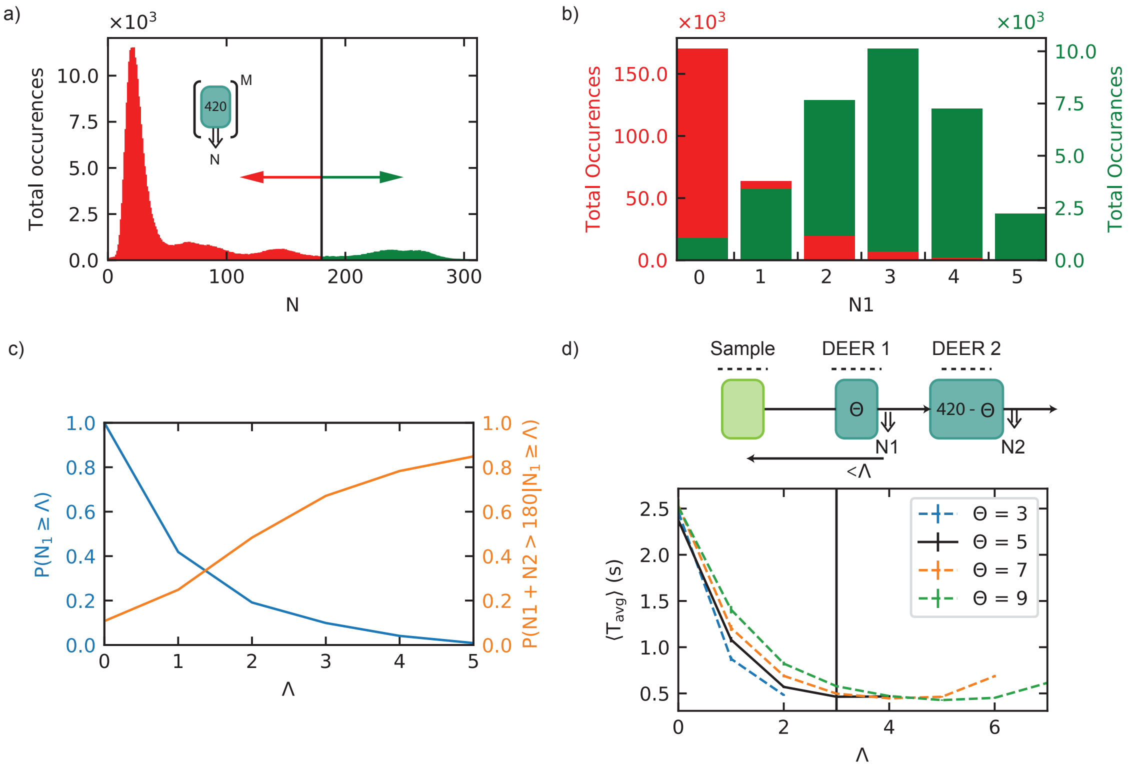

Supplementary Figure 7: Optimization to increase the experimental rate a) The distribution of outcomes for $K = 420$ DEER measurements. We define successful (unsuccessful) initializations as $N > 180$ ($N \leq 180$) indicated by the green (red). b) Distribution of outcomes after Θ DEER measurements, where as an example we take $\Theta = 5$. Green (red) bars correspond to the green (red) datasets in (a). c) The probability P(N1 $\geq \Lambda$) to measure an outcome N1 $\geq \Lambda$ after 5 DEER measurements (blue) and the probability P(N1 + N2 > 180|N1 $\geq \Lambda$) to obtain $N > 180$ after 420 DEER measurements, conditioned on having N1 $\geq \Lambda$ in the first 5 DEER measurements. d) The average time ($\langle T_{\mathrm{avg}} \rangle$) to complete one successful run of the method shown on top. A run is successful if N1 + N2 > 180. We calculate $\langle T_{\mathrm{avg}} \rangle$ as a function of Λ for different settings of Θ. We find a factor 5 increase in experimental rate for Θ = 5 and Λ = 3 compared to no thresholding (Λ = 0).

B. Active optical reset

In this section we implement the method devised above to increase the experimental rate. We use photoexcitation [5] to efficiently randomize/reset the P1 center states after failed initialisation attempts. This method results in a fourfold increase of experimental rate. We observe a trade-off for the laser power, with higher laser power decreasing the rate due to spectral diffusion and ionization of the NV [6] and increasing the rate due to resetting the nitrogen nuclear spin and JT configuration of the P1 bath, and find an optimal working point.

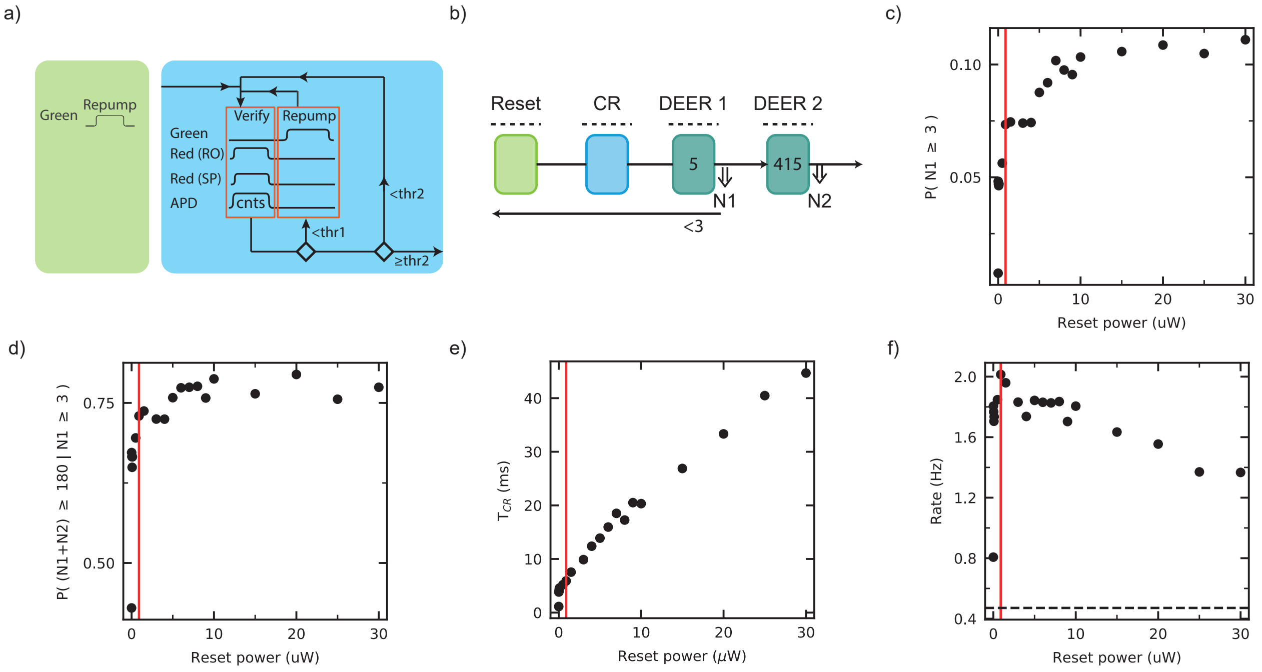

Supplementary Figure 8: **Active optical reset of P1 bath.** a) Illustration of the green (515 nm) optical reset pulse (left). Logical sequence showing the charge and resonance verification (CR) for the NV center (right). We count the number of photons (cnts) while resonantly exciting the NV center simultaneously at its readout (RO) and spin-pump (SP) transitions. If cnts $<$ thr1, the charge state is optically reset (repump, 30 μW). The experimental sequence is continued if cnts $\geq$ thr2 (thr2 $>$ thr1). If thr1 $\leq$ cnts $<$ thr2, a new verification step is entered. b) Schematic of the experimental sequence. An initial optical reset is performed (green, 5 μs) to excite the P1 bath [5]. Thereafter, the CR scheme (blue, see (a)) is implemented until the experimental sequence is continued. This scheme is followed by a DEER sequence (K=5, see main text). Based on the outcome of DEER 1 we either continue to DEER 2 (K=415) or go back to the start of the experimental sequence to apply an optical reset pulse. c),d) Plots of the probabilities to pass the desired threshold after DEER 1 and DEER 2, as a function of the optical reset pulse power. e) Average duration of the CR scheme (T_{CR}) as a function of optical reset pulse power. f) Experimental rate given the desired threshold (N1 + N2) $\geq$ 180. The dashed horizontal line marks the rate without optical reset pulse and without feedback based on the outcome N1. Vertical red lines in c),d),e),f) mark the reset power for maximal experimental rate.

Our experiments require that the NV centre is in the correct negative charge state and that its readout and spin-pump transitions are on resonance with the two 637 nm lasers. This is established by implementing a charge and resonance verification scheme (CR) [7]. Additional to this scheme, we use an optical pulse to reset the P1 center states. Supplementary Figs. 8a and b show the experimental sequence where the optical reset pulse is applied at the beginning. The CR scheme is thereafter implemented, followed by a short DEER sequence (DEER 1). At this point, feedback is implemented as the DEER 1 sequence provides information about the configuration of the P1 bath (see Supplementary Fig. 7b). Based on the method above, we apply an optical reset pulse if N1 $<$ 3. In Supplementary Figs. 8c,d), an increase of the probability of passing the desired thresholds for both DEER 1 and DEER 2 as a function of reset pulse power is shown. This demonstrates the reset pulse to be fully effective at a power of ∼10 μW.

We observe an increase of the duration of the CR scheme as a function of reset power (Supplementary Fig. 8e), likely due to spectral diffusion caused by photoexcitation of the P1 bath. The trade-off between optical reset and

increased CR scheme duration becomes apparent in Supplementary Fig. 8f, where the experimental rate is plotted as a function of optical reset power. The rate initially increases, followed by a decrease as the time required for CR verification becomes dominant. We find over a 4-fold increase of experimental rate at the optimal optical reset power of 0.9 µW. The observed increase in experimental rate here is close to theoretically predicted in the section above.

8. SUPPLEMENTARY NOTE: CORRELATION MEASUREMENTS FOR DIFFERENT JT AXES

In this section we provide the background for the correlation measurement on different JT axes (Fig. 2d, main text), and include an analysis of the complete dataset (additional to the selected data in the main text). We derive the expected correlation value C for a signal originating from a number n of P1 centers in such correlation measurements.

First, we define the regions of interest for the measurement outcomes in both JT axes ($N_{|+1,\mathrm{A}\rangle}$ and $N_{|+1,\mathrm{D}\rangle}$, see Supplementary Fig. 9). We fit the peaks in both distributions to Gaussian functions of the form $f = O + \sum_{q=1}^{q_{\mathrm{tot}}} A_q \cdot e^{-(N-N_{q,0})^2/2\cdot\sigma_{q,0}^2}$, where N corresponds to either $N_{|+1,\mathrm{A}\rangle}$ or $N_{|+1,\mathrm{D}\rangle}$ and q_{tot} is either 1 or 3. From these fits (black lines, Fig. 9), we obtain the FWHM of each Gaussian, and use these to define the areas of interest as shown in Supplementary Fig. 10. Note that these ranges for $N_{|+1,\mathrm{D}\rangle}$ ($N_{|+1,\mathrm{A}\rangle}$) are also used in experiments described in the main text and supplementary information for initialisation of S1, S2 and S3/S4 in $|+1,\mathrm{D}\rangle$ ($|+1,\mathrm{A}\rangle$), unless mentioned otherwise.

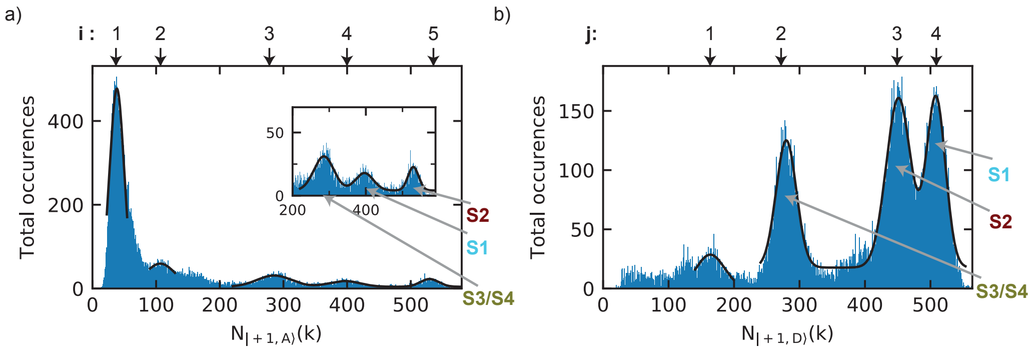

Supplementary Figure 9: Distributions of total occurrences. a) All data from the measurement of Fig. 2d in the main text. The histogram shows the total occurrences plotted as a function of outcome $\mathrm{N}_{|+1,\mathrm{A}\rangle}$ (irrespective of the outcome $\mathrm{N}_{|+1,\mathrm{D}\rangle}$). The black solid line corresponds to the fit. Numbers corresponding to an index, indicate the center of each fitted Gaussian. Inset: enlarged view of the same histogram indicating S2, S1 and S3/S4. b) Similar to (a), but for measurements on $|+1,\mathrm{D}\rangle$. The data in (b) is taken with an increased rate using the active optical reset method described in Supplementary Note 7, explaining why no peak at low counts is observed.

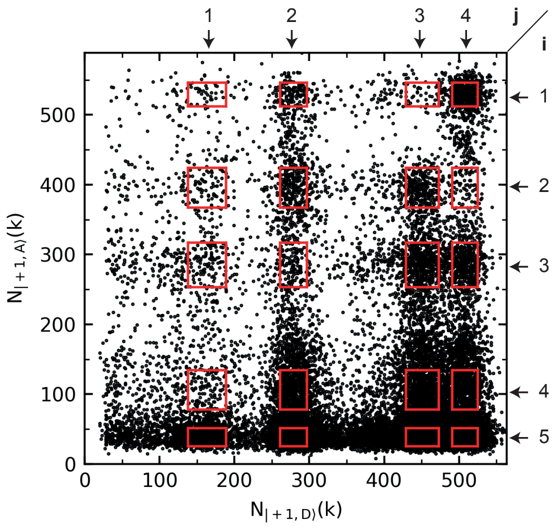

Supplementary Figure 10: **Complete dataset corresponding to the measurement in Fig. 2d (main text).** The red rectangular areas illustrate the regions defined by the FWHM as obtained from the fitted curves in Supplementary Fig. 9. We denote each area by indices (i,j). The correlation values C corresponding to these areas are shown in Supplementary Table 2.

Second, we define the required probability functions. For outcome $N_{|+1,\mathrm{D}\rangle}$, the probabilities for obtaining $N_{\mathrm{D}}^{\mathrm{min}} \leq N_{|+1,\mathrm{D}\rangle} \leq N_{\mathrm{D}}^{\mathrm{max}}$ irrespective of $N_{|+1,\mathrm{A}\rangle}$ are given by:

$$\mathrm{P}(N_{\mathrm{D}}^{\mathrm{min}} \leq N_{|+1,\mathrm{D}\rangle} \leq N_{\mathrm{D}}^{\mathrm{max}}) = \mathrm{P}_{\mathrm{D}}\big|_{\mathrm{min}}^{\mathrm{max}} = \int_{N_{\mathrm{D}}^{\mathrm{min}}}^{N_{\mathrm{D}}^{\mathrm{max}}} \mathrm{pdf}_{\mathrm{D}} \cdot dN_{|+1,\mathrm{D}\rangle}, \tag{22}$$

and, for outcome $N_{\mathrm{A}}^{\mathrm{min}} \leq N_{|+1,\mathrm{A}\rangle} \leq N_{\mathrm{A}}^{\mathrm{max}}$ irrespective of outcome $N_{|+1,\mathrm{D}\rangle}$, this is given as:

$$\mathrm{P}(N_{\mathrm{A}}^{\mathrm{min}} \leq N_{|+1,\mathrm{A}\rangle} \leq N_{\mathrm{A}}^{\mathrm{max}}) = \mathrm{P}_{\mathrm{A}}\big|_{\mathrm{min}}^{\mathrm{max}} = \int_{N_{\mathrm{A}}^{\mathrm{min}}}^{N_{\mathrm{A}}^{\mathrm{max}}} \mathrm{pdf}_{\mathrm{A}} \cdot dN_{|+1,\mathrm{A}\rangle}. \tag{23}$$

$\mathrm{pdf}_{\mathrm{D}}$ and $\mathrm{pdf}_{\mathrm{A}}$ are probability density functions. We additionally define a conditional probability

$$\mathrm{P}((N_{\mathrm{A}}^{\mathrm{min}} \leq N_{|+1,\mathrm{A}\rangle} \leq N_{\mathrm{A}}^{\mathrm{max}})|(N_{\mathrm{D}}^{\mathrm{min}} \leq N_{|+1,\mathrm{D}\rangle} \leq N_{\mathrm{D}}^{\mathrm{max}})) = \overline{\mathrm{P}_{\mathrm{A}}}\big|_{\mathrm{min}}^{\mathrm{max}} = \int_{N_{\mathrm{A}}^{\mathrm{min}}}^{N_{\mathrm{A}}^{\mathrm{max}}} \overline{\mathrm{pdf}_{\mathrm{A}}} \cdot dN_{|+1,\mathrm{A}\rangle} \tag{24}$$

with $\overline{\mathrm{pdf}_{\mathrm{A}}}$ the probability density function for outcomes $N_{|+1,\mathrm{A}\rangle}$ given $N_{\mathrm{D}}^{\mathrm{min}} \leq N_{|+1,\mathrm{D}\rangle} \leq N_{\mathrm{D}}^{\mathrm{max}}$.

Finally, we derive a bound for the correlation function C. Consider n P1 centers, each of which generates signal within $N_{\mathrm{D}}^{\mathrm{min}} \leq N_{|+1,\mathrm{D}\rangle} \leq N_{\mathrm{D}}^{\mathrm{max}}$ or within $N_{\mathrm{A}}^{\mathrm{min}} \leq N_{|+1,\mathrm{A}\rangle} \leq N_{\mathrm{A}}^{\mathrm{max}}$ if they are in the corresponding state. The correlation C for consecutive measurements then satisfies:

$$C = \frac{\overline{\mathrm{P}_{\mathrm{A}}}\big|_{\mathrm{min}}^{\mathrm{max}}}{\mathrm{P}_{\mathrm{A}}\big|_{\mathrm{min}}^{\mathrm{max}}} \geq \frac{n-1}{n}. \tag{25}$$

The inequality in Supplementary Eq. (25) is derived as follows. We consider k P1 centers, each with an identical probability p to be in any of the states $|m_I, i\rangle$ (here p is approximately 1/12). We consider two signal regions $N_{\mathrm{D}}^{\mathrm{min}} \leq N_{|+1,\mathrm{D}\rangle} \leq N_{\mathrm{D}}^{\mathrm{max}}$ and $N_{\mathrm{A}}^{\mathrm{min}} \leq N_{|+1,\mathrm{A}\rangle} \leq N_{\mathrm{A}}^{\mathrm{max}}$, and assume that there are n ($n \leq k$) P1 centers with a coupling that results in a signal in those regions. Note that no signal is generated in the respective region if more than one P1 center is simultaneously in the state $|+1, \mathrm{D}\rangle$ or $|+1, \mathrm{A}\rangle$, as the NV then accumulates a different phase ‡.

‡ For two P1 centers (out of S1-S4) that simultaneously occupy the same state, summation of coupling strengths and electron spin relaxation play a role for the acquired phase and are discussed in Supplementary Note 2C. Note also that the probability that two or more of the P1s considered here are in the same state simultaneously is small.

From the above, it follows that:

$$\mathrm{P_D}\big|_{\min}^{\max} = \mathrm{P_A}\big|_{\min}^{\max} = n \cdot p \cdot (1-p)^{k-1}. \tag{26}$$

The observation of a signal in the region $N_{\mathrm{D}}^{\min} \leq N_{|+1,\mathrm{D}\rangle} \leq N_{\mathrm{D}}^{\max}$ means that one of the n spins is in state $|+1,\mathrm{D}\rangle$ and thus conditional probability for the second measurement becomes:

$$\overline{\mathrm{P_A}}\big|_{\min}^{\max} = (n-1) \cdot p \cdot (1-p)^{k-2} = \frac{n-1}{n} \cdot \frac{1}{1-p} \cdot \mathrm{P_A}\big|_{\min}^{\max} \geq \frac{n-1}{n} \cdot \mathrm{P_A}\big|_{\min}^{\max}, \tag{27}$$

which yields Supplementary Eq. (25). In a similar way it follows that $\overline{\mathrm{P_A}}\big|_{\min}^{\max} \geq \mathrm{P_A}\big|_{\min}^{\max} \geq \frac{n-1}{n}\mathrm{P_A}\big|_{\min}^{\max}$, if the signal $N_{\mathrm{A}}^{\min} \leq N_{|+1,\mathrm{A}\rangle} \leq N_{\mathrm{A}}^{\max}$ is generated by m spins that are not necessarily the same as the n spins that generate signal $N_{\mathrm{D}}^{\min} \leq N_{|+1,\mathrm{D}\rangle} \leq N_{\mathrm{D}}^{\max}$.

The values of C obtained for all areas as indicated in Supplementary Fig. 10 (red rectangles) are shown in Supplementary Table 2. For the areas (i,j) = (1,3) and (2,4) (as in Fig. 2d in the main text) we obtain C = 0.22(4) and 0.40(5) respectively, which indicates a single spin. For (i,j) = (3,4) (as in Fig. 2d main text), C = 0.47(4) indicating 1 or 2 spins. For areas such as (i,j) = (5,1), (5,2), (5,3) and (5,4) we find C ≈ 1 indicating a large number of spins n. For these areas one would indeed expect a larger number of spins because they correspond to weaker dipolar coupling to the NV. Interestingly, some areas have a C value that is significantly above unity such as (1,4), (2,1) and (2,2). For the area (1,4) the highest value is observed (C = 2.2(2)), this suggests that there might be preferred combinations of $|m_i, i\rangle$ states for S1 and S2.

i\j	1	2	3	4
1	1.0(2)	1.0(1)	0.22(4)	2.2(2)
2	1.6(2)	1.5(1)	1.20(8)	0.40(5)
3	1.4(1)	0.47(4)	1.14(6)	1.15(6)
4	0.74(7)	1.17(5)	1.0(1)	0.94(4)
5	0.99(4)	0.94(2)	1.00(2)	0.97(2)

Supplementary Table 2: C values for areas as shown in Supplementary Fig. 10

9. SUPPLEMENTARY NOTE: NV-P1 DIPOLAR COUPLING SIGN IN $|+1,\mathrm{A}\rangle$

This section demonstrates coherent control of the ^{14}N spin and extracts the sign of the NV-P1 dipolar coupling for the electron spin of S1, S2 and S3/S4 in the $|+1,\mathrm{A}\rangle$ state (similar as in Fig. 4b of the main text for $|+1,\mathrm{D}\rangle$). First, we choose an initialisation sequence with a low K (K=6), which with high probability prepares either S1, S2 or S3/S4 in $|+1,\mathrm{A}\rangle$. Then we calibrate a π pulse that implements a CNOT between the electron (control) and nitrogen nuclear spin (target) for a P1 in the $|+1,\mathrm{A}\rangle$ state, see Supplementary Fig. 11a. This pulse is only resonant with the $m_I = +1 \leftrightarrow 0$ transition, if the P1 electron spin is in the $|\uparrow\rangle$ state.

We use the same experimental procedure as in the main text for $|+1,\mathrm{D}\rangle$, to determine the NV-P1 coupling signs (Supplementary Fig. 11b). The value R is defined as:

$$R = \frac{P_{(+y)} - P_{(-y)}}{P_{(+y)} + P_{(-y)}}, \tag{28}$$

with $P_{(+y)}$ ($P_{(-y)}$) the probability that the outcome of the DEER readout sequence is within the FWHM range corresponding to S1, S2 or S3/S4 (see Supplementary Note 8), and the subscript +y (-y) indicates the readout basis of the DEER(y) sequence used to initialize the electron spin. The result shows that spin S1 and S3/S4 have a positive coupling sign, whereas it is negative for S2. Note that S1, S2 and S3/S4 have the same NV coupling sign in $|+1,\mathrm{A}\rangle$ and $|+1,\mathrm{D}\rangle$.

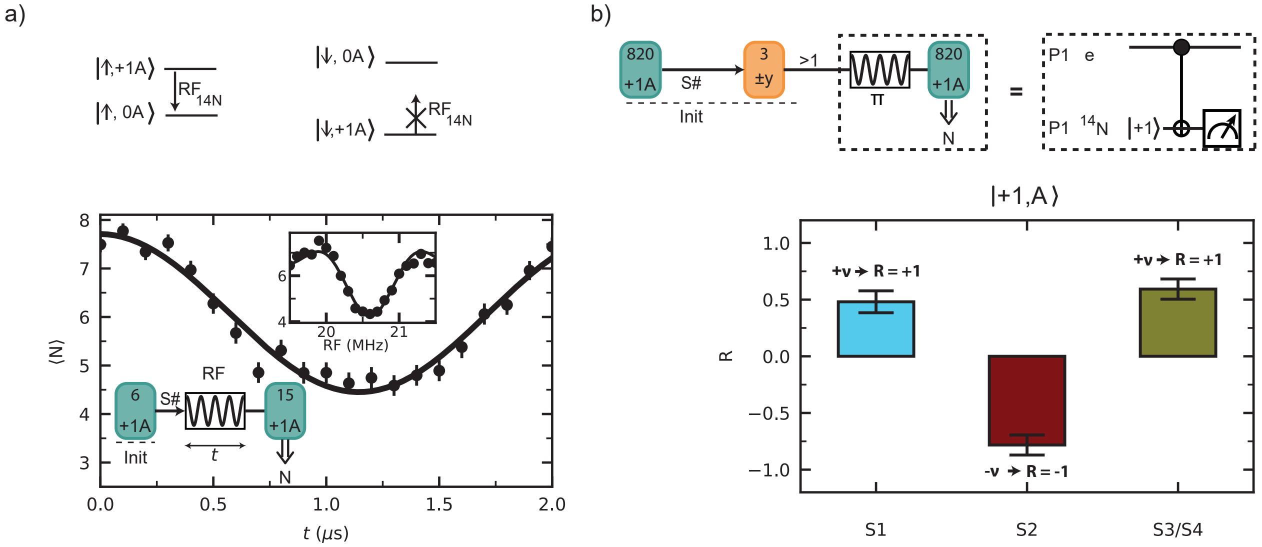

Supplementary Figure 11: **Coupling sign of NV-P1 dipolar interaction for different P1 centers in the $|+1, \mathrm{A}\rangle$ state.** a) Top: energy level structure of the P1 electron spin in the $\{0\mathrm{A}, +1\mathrm{A}\}$ subspace. Bottom: A short initialisation sequence (K=6) prepares either S1, S2 or S3/S4 in $|+1, \mathrm{A}\rangle$ (without knowledge of which P1 is prepared in which run) and the length t of a pulse at frequency $\mathrm{RF} = \mathrm{RF}_{14\mathrm{N}} = 20.55$ MHz is varied. The nitrogen nuclear spin is driven conditionally on the electron spin state (top). Inset: NMR spectrum obtained by varying the frequency (RF) for a fixed pulse duration t. b) Value of R (see Methods) measured for the P1s in $|+1, \mathrm{A}\rangle$. Positive values correspond to a positive coupling sign.

10. SUPPLEMENTARY NOTE: NV-P1 COUPLING IN $|+1, \mathrm{A}\rangle$

In Fig. 3a of the main text the effective NV-P1 dipolar coupling (Supplementary Eq. (13)) for S1, S2, S3/S4 in $|+1, \mathrm{D}\rangle$ is measured. Here, we measure the effective dipolar coupling of these spins in $|+1, \mathrm{A}\rangle$, see Supplementary Fig. 12. We initialize each P1 center by setting the requirement that the outcome of the DEER initialisation sequence is within the range for $|+1, \mathrm{A}\rangle$ (see Supplementary Fig. 9a).

We obtain effective dipolar couplings ν of $2\pi \cdot 1.35(2)$, $2\pi \cdot 2.006(9)$ and $2\pi \cdot 1.06(2)$ kHz for S1, S2 and S3/S4 respectively, using the fit function in eq. 11 (see Methods). A comparison with the couplings for $|+1, \mathrm{D}\rangle$ (Fig. 3a in the main text) shows that the effective NV-P1 dipolar coupling strength differs for the two JT axes (see section 3 for the theoretical treatment).

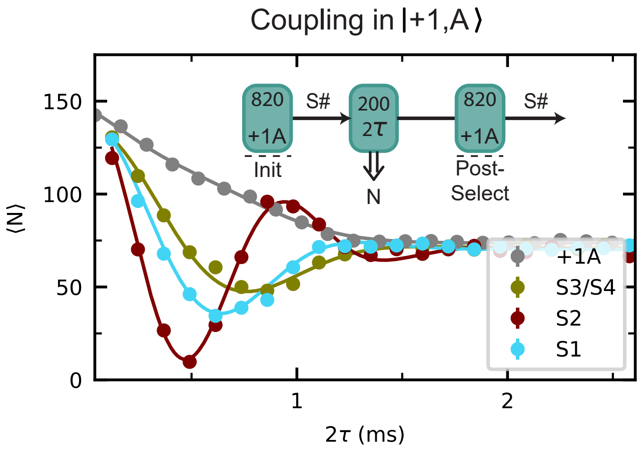

Supplementary Figure 12: **Measuring NV-P1 coupling strength in $|+1, \mathrm{A}\rangle$.** We initialize S1, S2 or S3/S4 in $|+1, \mathrm{A}\rangle$ and vary the interaction time 2τ of a DEER sequence (K=200). To improve the signal, the results are post-selected on again obtaining $|+1, \mathrm{A}\rangle$. Inset: experimental sequence. Grey: without P1 initialisation (data from Fig. 1c main text).

11. SUPPLEMENTARY NOTE: MAGNETIC FIELD STABILITY

Here we describe the magnetic field stabilisation used during the Ramsey and entanglement experiments in this manuscript, as well as during the data in Supplementary Fig. 2b. We use three permanent magnets on motorized linear translation stages to create a static magnetic field. To compensate for magnetic field fluctuations we repeatedly measure the NV $|0\rangle \leftrightarrow |-1\rangle$ frequency (f_{NV}) and this signal is fed back to one of the magnet stages until (f_t - 1.5 kHz $\leq f_{\mathrm{NV}} \leq f_t$ + 1.5 kHz), with target frequency $f_t = 2.749692$ GHz. We interleave this protocol (typically every 20 minutes) with repetitions of an experimental sequence.

Supplementary Fig. 13a shows the time trace of f_{NV} before and after interleaved experiments. The two red lines indicate $f_t \pm 1.5$ kHz. The magnetic field can freely drift during the experimental sequences, but right before starting the next run the field is stabilized. From the histogram we infer that on average f_{NV} fluctuates with $\sigma = 4.07$ kHz around $f = 2.749690$ GHz during feedback.

To monitor the complete magnetic field vector $\mathbf{B} = (B_x, B_y, B_z)$ during experiments with the stabilization protocol interleaved, we measure $f_{+1\mathrm{A}}, f_{+1\mathrm{B}}, f_{+1\mathrm{C}}$ and $f_{+1\mathrm{D}}$ (see Fig. 1b main text). In a similar approach as in section 4, we fit a parabola to the four frequencies and use the residuals consisting of the difference of the four fitted frequencies and the corresponding frequencies obtained from Supplementary Eq. (1) to perform a least-squares minimization to find $\mathbf{B}$.

The obtained values for B_x, B_y and B_z are shown in Figs. 13b,c,d. The standard deviations of these histogram verify a magnetic field stability during this protocol with $\sigma_{B_x} = 20$ mG, $\sigma_{B_y} = 16$ mG and $\sigma_{B_z} = 3$ mG. Note that the average values of $\mathbf{B}$ from Supplementary Fig. 13 show a discrepancy with the values obtained from the fit in the main text and section 4, indicating that these measurements have been taken under slightly different conditions.

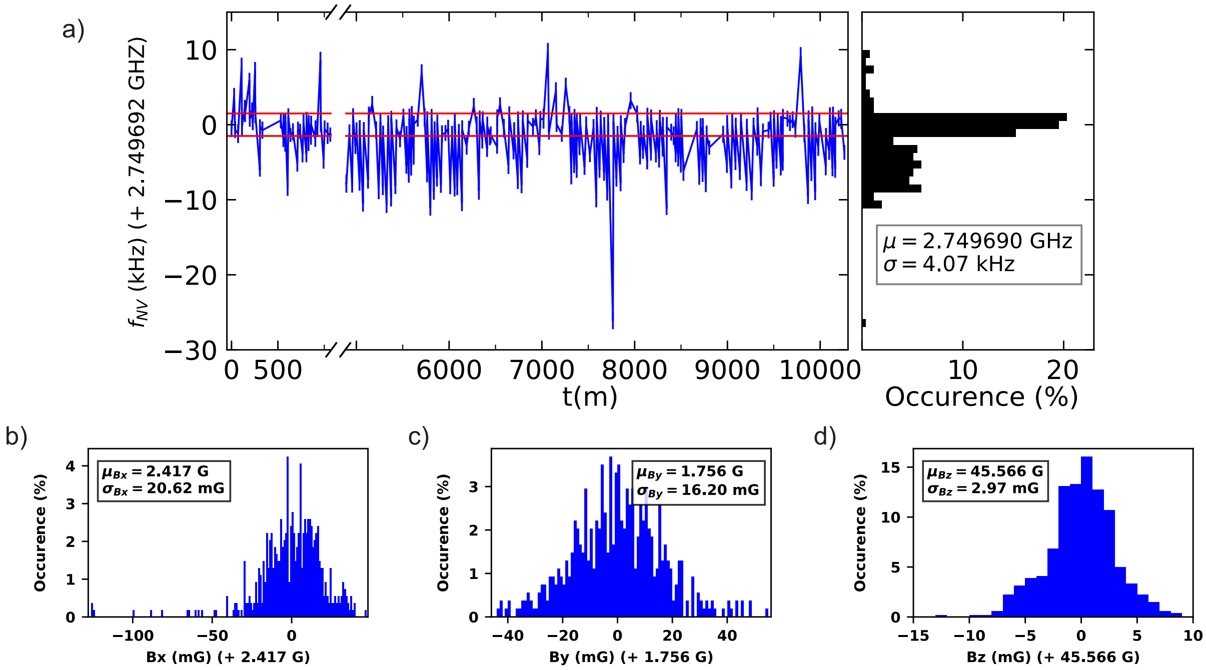

Supplementary Figure 13: **Magnetic field stability.** (a) Time trace of all measured NV resonance frequencies before and after performing repetitions of an experimental sequence. The red lines indicate the region (± 1.5 kHz) to within which we stabilize before each measurement. (b,c,d) Distribution of measured B_x, B_y and B_z during 220 hours, while stabilizing f_{NV}.

12. SUPPLEMENTARY NOTE: COHERENCE TIMES OF S3/S4 IN $|{+1}, \mathrm{D}\rangle$

We use the same experimental sequence as in Fig. 5 of the main text to measure the T_1, T_2^* and T_2 of the electron and nitrogen nuclear spin of S3/S4 (see Supplementary Fig. 14a). The observed coherence times are presented in Supplementary Table 3, where also the coherence times of S1/S2 as measured in the main text are shown for completeness.

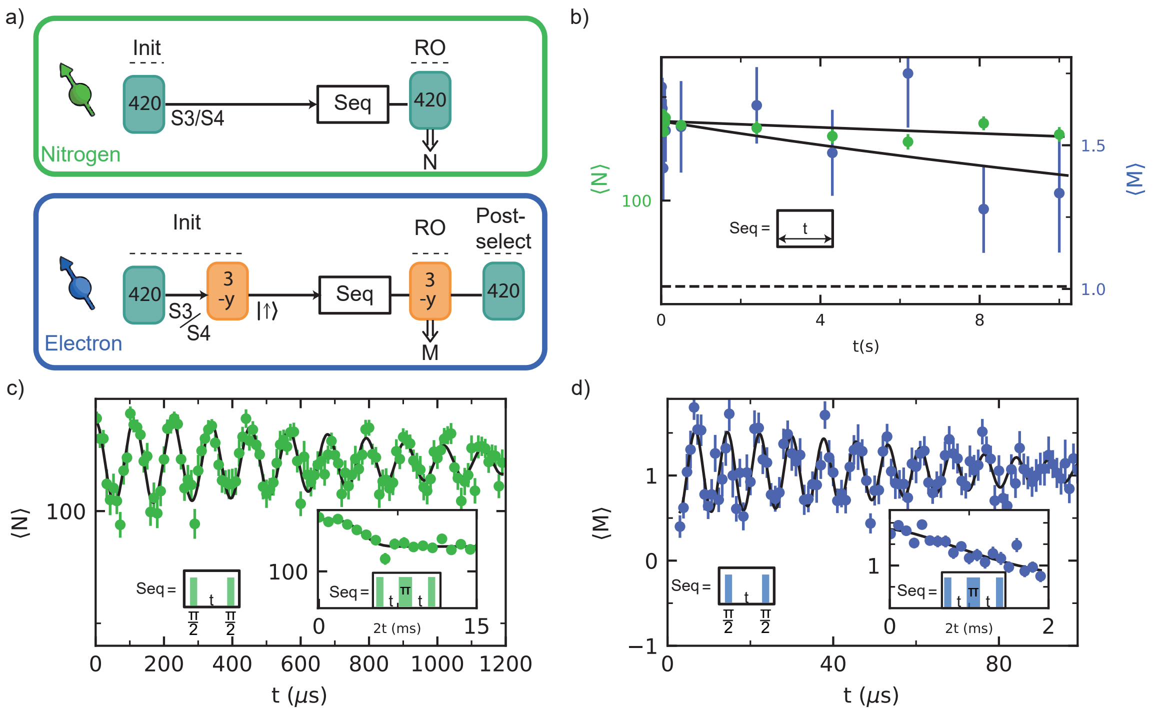

Supplementary Figure 14: **Timescales S3/S4.** (a) Sequence for initialisation of S3/S4 in $|{+1}, \mathrm{D}\rangle$ (top) and sequence for initialisation of all degrees of freedom of S3/S4 (bottom). These sequences are used in b,c and d. b) Relaxation of a combination of: the nitrogen nuclear spin state, JT axis and charge state (green), and only the electron spin state (blue). We fit (solid lines) both curves to $o + A_0 e^{-t/T}$. c) Ramsey and spin echo (inset) experiments on the nitrogen nuclear spin. d) Ramsey and spin echo (inset) experiments on the electron spin. The obtained coherence times are shown in Supplementary Table 3. See Data analysis (Methods) for the complete fit functions of the Ramsey (Eq. 11) and spin echo (Eq. 12) experiments presented in this figure.

	S3/S4	S1/S2
$T_{\lvert+1,D\rangle}$	104(38) s	40(4) s
$T_{1\mathrm{e}}$	26(20) s	21(7) s
$T_{2\mathrm{e}}^*$	82(10) µs	50(3) µs
$T_{2\mathrm{N}}^*$	1.06(9) ms	0.201(9) ms
$T_{2\mathrm{e}}$	1.5(1.2) ms	1.00(4) ms
$T_{2\mathrm{N}}$	4.5(4) ms	4.2(2) ms

Supplementary Table 3: Measured coherence and relaxation times of S3/S4 and S1/S2. For experimental data, see Supplementary Fig. 14 and main text Fig. 5 respectively.

13. SUPPLEMENTARY NOTE: EFFECTIVE GYROMAGNETIC RATIO AND SPIN COHERENCE

Here we consider the effect of the electron-nuclear spin mixing due to a relatively large perpendicular hyperfine component ($\gamma_e|\mathbf{B}| \sim A_\perp$) on the expected coherence times. First we calculate the effective gyromagnetic ratios (labelled $\gamma_{n_{\mathrm{eff}}}$ and $\gamma_{e_{\mathrm{eff}}}$) of the two transitions used in the experiments of Fig. 5c, d (main text). We consider the six energy levels of a single P1 center distorted along the JT axis D at the experimental magnetic field, see dashed line

Supplementary Fig. 15. Subsequently, we investigate the susceptibility of the energy levels to each component (B_i) of the magnetic field vector ($\mathbf{B}$). We then determine the tangent of both energy levels connected to the green (blue) double headed arrows at the experimental value of B_i (see Supplementary Fig. 15) and calculate $\gamma_{n_{\mathrm{eff}},i}$ ($\gamma_{e_{\mathrm{eff}},i}$) as the difference between two tangents. The green (blue) arrow indicates the transition used in Fig. 5c(d) in the main text. We find $\gamma_{n_{\mathrm{eff}},z}$ ($\gamma_{e_{\mathrm{eff}},z}$) is more than 10 times larger than $\gamma_{n_{\mathrm{eff}},x/y}$ ($\gamma_{e_{\mathrm{eff}},x/y}$), and therefore we will only consider $\gamma_{n_{\mathrm{eff}},z}$ and $\gamma_{e_{\mathrm{eff}},z}$.

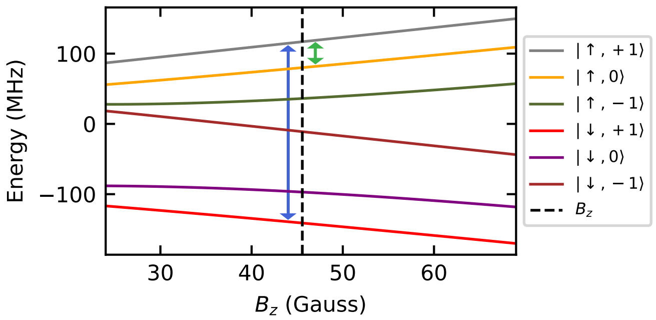

Supplementary Figure 15: **Energy levels for a single P1 center in JT axis D**. Simulation of the six energy levels which are labelled according to their P1 electron and nitrogen nuclear spin state. The black dashed line denotes the experimental magnetic field value. The green (blue) double headed arrow indicates the transition used in the nitrogen (electron) coherence experiments (Fig. 5c,d)

With the approach above, we obtain $\gamma_{n_{\mathrm{eff}},z} = 2\pi\cdot$ 0.206 MHz/G ($\sim 700\gamma_n$) and $\gamma_{e_{\mathrm{eff}},z} = 2\pi\cdot$ 2.60 MHz/G ($\sim 0.93\gamma_e$). Defining $\Gamma = \gamma_{e_{\mathrm{eff}},z}/\gamma_{n_{\mathrm{eff}},z}$, we find $\Gamma = 12.6$. Thus, based on the spin mixing, we would expect the nitrogen nuclear spin coherence to be a factor 12.6 larger than the electron spin coherence. For S3/S4, we find a ratio $T^*_{2\mathrm{N}}/T^*_{2\mathrm{e}} \sim 12$ between the nitrogen nuclear spin and electron coherence (see Supplementary Table 3), which agrees well. However, for spin S1 and S2 we find $T^*_{2\mathrm{N}}/T^*_{2\mathrm{e}} \sim 4$. The remaining discrepancy by a factor 3 is not yet understood.

14. SUPPLEMENTARY NOTE: ENTANGLEMENT SEQUENCE

In this section the entanglement generation sequence for S1 and S2 is explained in detail. We begin by initializing S1 and S2 in $|\Psi\rangle_{\mathrm{init}} = |\uparrow,+1,\mathrm{D}\rangle_{\mathrm{S1}}\ |\downarrow,+1,\mathrm{A}\rangle_{\mathrm{S2}} = |\uparrow\downarrow\rangle$. Next we apply $U_1 = R_x(\pi/2)_{\mathrm{S1}}R_x(\pi/2)_{\mathrm{S2}}$, consisting of two $\frac{\pi}{2}$ pulses along x to obtain the state:

$$U_1\,|\Psi\rangle_{\mathrm{init}} = \frac{1}{2}\big(-i\,|\uparrow\uparrow\rangle + |\uparrow\downarrow\rangle - |\downarrow\uparrow\rangle - i\,|\downarrow\downarrow\rangle\big) \tag{29}$$

This state evolves for time t and accumulates phase due to dipolar coupling J, as $U_{zz}(t) = e^{-i\cdot J\cdot S_z S_z t}$:

$$U_{zz}U_1\,|\Psi\rangle_{\mathrm{init}} = \frac{1}{2}e^{i\cdot J\cdot t/4}\big(-i\cdot e^{-i\cdot J\cdot t/2}\,|\uparrow\uparrow\rangle + |\uparrow\downarrow\rangle - |\downarrow\uparrow\rangle - i\cdot e^{-i\cdot J\cdot t/2}\,|\downarrow\downarrow\rangle\big) \tag{30}$$

Thereafter, we apply $U_2 = R_x(\pi)_{\mathrm{S1}}R_x(\pi)_{\mathrm{S2}}$, consisting of two π pulses along X, followed by another free evolution time t:

$$|\Psi\rangle_{\mathrm{final}} = U_{zz}U_2U_{zz}U_1\,|\Psi\rangle = \frac{1}{2}e^{i\cdot J\cdot t/2}\big(i\cdot e^{-i\cdot J\cdot t}\,|\uparrow\uparrow\rangle + |\uparrow\downarrow\rangle - |\downarrow\uparrow\rangle + i\cdot e^{-i\cdot J\cdot t}\,|\downarrow\downarrow\rangle\big) \tag{31}$$

Rewriting $|S2\rangle$ in the X basis, where $|+\rangle = \frac{|\uparrow\rangle+|\downarrow\rangle}{\sqrt{2}}$ and $|-\rangle = \frac{|\uparrow\rangle-|\downarrow\rangle}{\sqrt{2}}$, results in:

$$|\Psi\rangle_{\mathrm{final}} = \tfrac{1}{2}e^{i\cdot J\cdot t/2}\left[\left(\frac{i\cdot e^{-i\cdot J\cdot t}+1}{\sqrt{2}}\right)|\uparrow +\rangle + \left(\frac{i\cdot e^{-i\cdot J\cdot t}-1}{\sqrt{2}}\right)|\uparrow -\rangle + \left(\frac{i\cdot e^{-i\cdot J\cdot t}-1}{\sqrt{2}}\right)|\downarrow +\rangle + \left(\frac{-i\cdot e^{-i\cdot J\cdot t}-1}{\sqrt{2}}\right)|\downarrow -\rangle\right] \tag{32}$$

Note that at time $2t = \frac{\pi}{|J|}$, and a negative coupling J, this yields to the entangled state:

$$|\Psi\rangle_{\mathrm{final}} = -e^{\frac{-i\pi}{4}}\,\frac{|\uparrow -\rangle + |\downarrow +\rangle}{\sqrt{2}} \tag{33}$$

15. SUPPLEMENTARY NOTE: OPTIMIZATION OF INITIALISATION/READOUT

A. ^{14}N and JT state

Here we explain the optimization of single-shot readout and initialisation of the ^{14}N and JT state of individual P1 centers. We define the fidelity of initialisation of S1 in $|{+1}, \mathrm{D}\rangle$ and S2, S3/S4 not in that state as

$$F_{\mathrm{S1}} = \mathrm{P}(\mathrm{N}(k+1) > N_{\mathrm{RO}} | \mathrm{N}(k) > N_{\mathrm{S1}}), \tag{34}$$

whereas for a mixture of all other possibilities we define

$$F_{\mathrm{notS1}} = \mathrm{P}(\mathrm{N}(k+1) \leq N_{\mathrm{RO}} | \mathrm{N}(k) \leq N_{\mathrm{notS1}}). \tag{35}$$

In both cases $\mathrm{P}(X|Y)$ is the probability to obtain X given Y. We define the combined initialisation and readout fidelity of these two cases as:

$$F = \frac{F_{\mathrm{S1}} + F_{\mathrm{notS1}}}{2}. \tag{36}$$

The correlation plot between two subsequent measurements of $K = 820$ binned DEER sequences is shown in Supplementary Fig. 16a. First, we find a threshold that separates S1 from other P1 centers by setting $N_{\mathrm{S1}} = N_{\mathrm{notS1}} = N_{\mathrm{RO}}$ all equal to N_{sweep} and calculate the combined initialisation and readout fidelity F while we sweep this parameter. A local maximum $N_{\mathrm{opt}} = 477$ is found separating spins S1 and S2 as shown in Supplementary Fig. 16b (red dashed line). Second, we set $N_{\mathrm{RO}} = N_{\mathrm{notS1}} = N_{\mathrm{opt}}$ to distinguish between S1 and not S1 and vary N_{S1} as shown in Supplementary Fig. 16c. As a trade-off between the success rate and fidelity F we choose $N_{\mathrm{S1}} = 522$ to maintain a success probability > 0.2. For Fig. 2f in the main text we have thus used $N_{\mathrm{notS1}} = N_{\mathrm{RO}} = 477$ and $N_{\mathrm{S1}} = 522$.

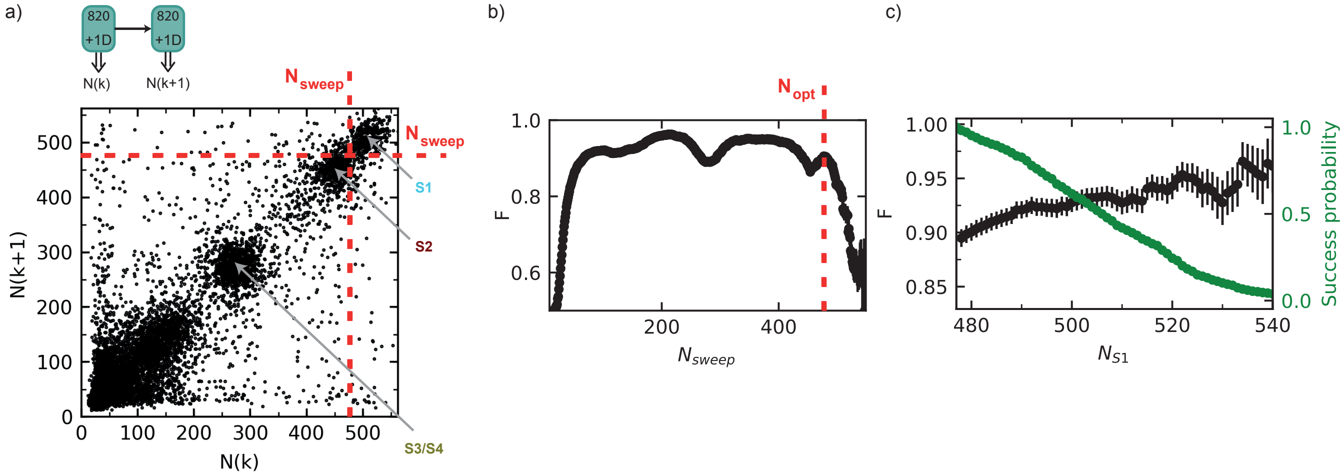

Supplementary Figure 16: **Optimization of combined initialisation and readout of S1 in $|{+1}, \mathrm{D}\rangle$. a)** Full correlation plot for consecutive measurement outcomes $\mathrm{N}(k)$ and $\mathrm{N}(k+1)$, both for $|{+1}, \mathrm{D}\rangle$ (same dataset as Fig. 2e in the main text). We set the thresholds $N_{\mathrm{S1}} = N_{\mathrm{notS1}} = N_{\mathrm{RO}}$ all equal to N_{sweep} (see main text Fig. 2e). Using these thresholds we calculate F as in Supplementary Eq. (36) for different values of N_{sweep}. **b)** Fidelity F as a function of N_{sweep}. The red dashed line indicates a local maximum of F (here $N_{\mathrm{sweep}} = N_{opt} = 477$) that optimally separates between S1 and S2 under the given constraints ($N_{\mathrm{S1}} = N_{\mathrm{notS1}} = N_{\mathrm{RO}} = N_{\mathrm{sweep}}$). The same value ($N_{\mathrm{sweep}} = N_{\mathrm{opt}}$) is shown by the red dashed lines in (a). **c)** Further improvement of F at the cost of experimental rate is achieved by a stricter initialisation threshold N_{S1}. First we set $N_{\mathrm{notS1}} = N_{\mathrm{RO}} = N_{\mathrm{opt}}$ and vary N_{S1}. The fidelity F is plotted as function of N_{S1} (black). The success probability of initialisation of S1 in $|{+1}, \mathrm{D}\rangle$ as compared to when $N_{\mathrm{S1}} = 477$ is plotted in green as a function of N_{S1}.

B. Electron spin initialisation and readout

We initialize and measure the electron spin state of P1 centers through a DEER(y) sequence following initialisation of the $|{+1}, \mathrm{D}\rangle$ or $|{+1}, \mathrm{A}\rangle$ state. Again, we use the correlation of consecutive measurements $\mathrm{M}(k)$ and $\mathrm{M}(k+1)$ to determine the combined initialisation and readout fidelity. First, we define the fidelity of preparing $|{\uparrow}\rangle$ as

$$F_{|\uparrow\rangle} = \mathrm{P}(\mathrm{M}(k+1) > M_{\mathrm{RO}} | \mathrm{M}(k) > M_{|\uparrow\rangle}), \tag{37}$$

and the fidelity of preparing $|\downarrow\rangle$ as

$$F_{|\downarrow\rangle} = \mathrm{P}(\mathrm{M}(k+1) \leq M_{\mathrm{RO}} | \mathrm{M}(k) \leq M_{|\downarrow\rangle}). \tag{38}$$

Again, the combined initialisation and readout fidelity is given as

$$F_{|\uparrow\rangle/|\downarrow\rangle} = \frac{F_{|\uparrow\rangle} + F_{|\downarrow\rangle}}{2}. \tag{39}$$

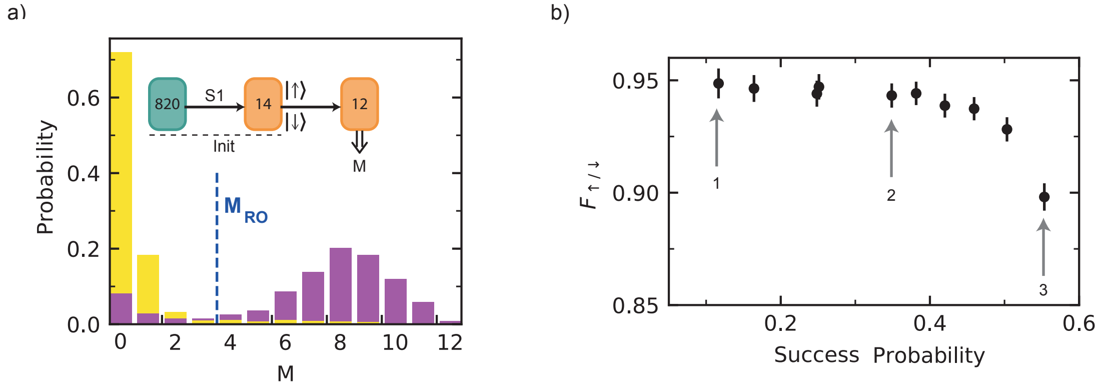

Supplementary Figure 17: **Optimization of combined electron initialisation and readout fidelity.** We trade off fidelity and experimental success rate by setting the thresholds and bin sizes of the DEER(y) sequences used. For optimization, a large range of thresholds and bin sizes are explored. **a)** An exemplary case of probability distributions after initialisation. Here, after preparation of S1 in $|+1, \mathrm{D}\rangle$, the electron spin is initialized through a DEER(y) (L = 14) with thresholds $M_{|\uparrow\rangle}$ (> 1) and $M_{|\downarrow\rangle}$ ($\leq$1) before reading out (L = 12). The parameters in this exemplary case ensure a larger success probability ($\approx$ 0.55) but lower $F_{|\uparrow\rangle/|\downarrow\rangle}$ = 0.90(1) as compared to those in Fig. 3d of the main text. **b)** The fidelity $F_{|\uparrow\rangle/|\downarrow\rangle}$ against the success probability of initialisation in $|\uparrow\rangle$. We calculate $F_{|\uparrow\rangle/|\downarrow\rangle}$ for a range of initialisation and readout bin sizes (L) and thresholds $M_{|\downarrow\rangle}$, $M_{|\uparrow\rangle}$ and M_{RO}. Only the maximum fidelities are shown, grouped over 10 intervals between 0.1 and 0.6 success probability. To ensure enough statistics, we only include intervals above 0.1 success probability. Numbered arrows indicate example cases: (1) parameters and probability histogram as shown in Fig. 3d in the main text; (2) L = 14 for initialisation, L = 8 for readout, $M_{|\uparrow\rangle}$ (> 8), $M_{|\downarrow\rangle}$ ($\leq$1) and M_{RO} (= 2); (3) corresponds to panel (a).

We calculate $F_{|\uparrow\rangle/|\downarrow\rangle}$ for a large range of initialisation and readout bin sizes (L) and thresholds ($M_{|\downarrow\rangle}$, $M_{|\uparrow\rangle}$ and M_{RO}). In Supplementary Fig. 17b maximum values of $F_{|\uparrow\rangle/|\downarrow\rangle}$ are shown as a function of success probability. We trade-off $F_{|\uparrow\rangle/|\downarrow\rangle}$ against success probability to obtain a combined initialisation and readout fidelity of $F_{|\uparrow\rangle/|\downarrow\rangle} = 0.95(1)$ while maintaining a success probability above 0.1.

C. Optimization of sequential initialisation in $|+1, \mathrm{D}\rangle$ and $|+1, \mathrm{A}\rangle$

For entanglement generations of S1 and S2 we optimize for high total initialisation and readout fidelity while maintaining a fast experimental rate. To initialize the P1 centers in the desired $|m_I, i\rangle$ state, we use 4 sequential steps with real time logic. Firstly we use $K = 5$ DEER measurements on $|+1, \mathrm{A}\rangle$ to quickly assess the likelihood of S2 in this state. If the outcome is below a threshold ($N_{|+1,\mathrm{A}\rangle} = 3$), we apply fast logic: we abort the sequence, apply an optical reset (Supplementary Note 7) and start again. If the threshold is passed, we continue. Secondly we repeat this on $|+1, \mathrm{D}\rangle$ to assess the likelihood of S1 in this state. If successful, we continue with DEER repetitions on $|+1, \mathrm{D}\rangle$ and perform a third step after $K = 22$ to distinguish S1 from S3/S4. To finish the initialization of S1, we continue to K = 820, required to distinguish S1 from S2. Fourthly, we apply DEER sequences on $|+1, \mathrm{A}\rangle$ and hereafter implement fast logic. With S1 initialized in $|+1, \mathrm{D}\rangle$, S2 is more easily distinguished from S3/S4. Therefore we perform the logic after $K = 50$ DEER sequences. Because consecutive measurements can disturb earlier prepared states, the small number of repetitions for the initialisation of S2 is beneficial for the overall fidelity. The probability to have both P1 centers in the desired state is small, and this limits the experimental rate. By heralding and the use of subsequent fast logic, we significantly speed up because the initialization of S2 is not attempted if the initialization of S1 fails.

After initialisation of the JT and nitrogen state, we perform electron spin initialisation. For electron spin initialisation and readout we optimize in a similar way as done in Subsection 15 B. However, we now introduce additional measurements in order to take disturbance due to sequential measurements into account and find a best combined initialisation and readout fidelity for both spins. Finally, we optimize the sequential initialisation (S1 in $|\uparrow\rangle$ then S2 in $|\downarrow\rangle$) for a high rate and fidelity but minimal disturbance of the initialized state of the other P1 center.

16. SUPPLEMENTARY NOTE: NV FLUORESCENCE RATE REFERENCE

In this section, we verify that the discrete jumps in the DEER time traces (Fig. 2a of the main text) are not due to the changes in the detected fluorescence rate of the NV itself (e.g. due to ionization, spatial or spectral drifts). As shown in Supplementary Fig. 18, we repeatedly apply the DEER sequence while alternating between opposite readout bases. We combine these measurements to obtain a continuous DEER signal and a reference of the detected NV fluorescence rate. The DEER signal shows discrete jumps. These jumps are not observed in the reference signal, thus excluding that they are caused by changes in the detected NV fluorescence rate.

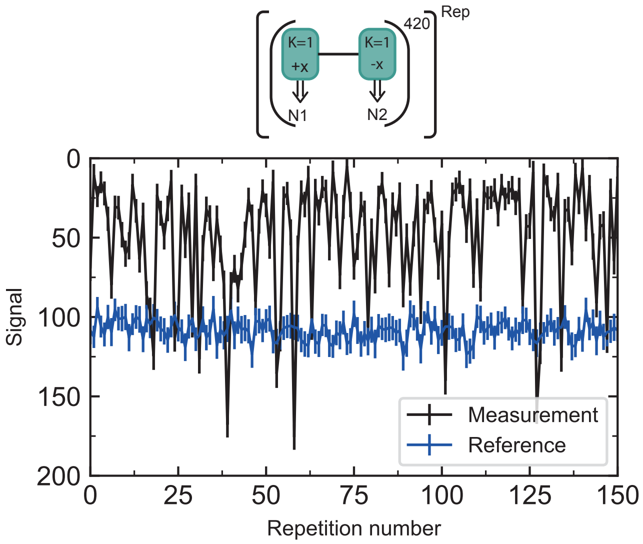

Supplementary Figure 18: **Continuous DEER signal with reference signal.** Top: Experiment in which we alternate between a single DEER sequence (K=1, $f_{+1\mathrm{D}}$ as in main text Fig. 2b) with phase $+x$ and one with $-x$ for the final $\pi/2$ pulse. The outcomes N_1 and N_2 are summed in a bin size of 420 to obtain N_{bin1} and N_{bin2}. The reference signal for the NV fluorescence detection rate is defined as $N_{\mathrm{ref}} = (N_{\mathrm{bin1}}+N_{\mathrm{bin2}})/2$. The measurement signal is given as $(2\langle N_{\mathrm{ref}}\rangle$ - N_{bin1} + $N_{\mathrm{bin2}})/2$, here $\langle N_{\mathrm{ref}}\rangle$ is the mean over the full dataset. Bottom: the measurement signal shows similar discrete jumps as in Fig. 2a of the main text, while the reference measurement remains approximately constant over time.

[1] Smith, W., Sorokin, P., Gelles, I. & Lasher, G. Electron-spin resonance of nitrogen donors in diamond. *Phys. Rev.* **115**, 1546 (1959).

[2] Knowles, H. S., Kara, D. M. & Atatüre, M. Observing bulk diamond spin coherence in high-purity nanodiamonds. *Nat. Mater.* **13**, 21–25 (2014).

[3] De Lange, G., Wang, Z., Riste, D., Dobrovitski, V. & Hanson, R. Universal dynamical decoupling of a single solid-state spin from a spin bath. *Science* **330**, 60–63 (2010).

[4] De Lange, G. *et al.* Controlling the quantum dynamics of a mesoscopic spin bath in diamond. *Sci. Rep.* **2**, 1–5 (2012).

[5] Heremans, F., Fuchs, G., Wang, C., Hanson, R. & Awschalom, D. Generation and transport of photoexcited electrons in single-crystal diamond. *Appl. Phys. Lett.* **94**, 152102 (2009).

[6] Robledo, L., Bernien, H., Van Weperen, I. & Hanson, R. Control and coherence of the optical transition of single nitrogen vacancy centers in diamond. *Phys. Rev. Lett.* **105**, 177403 (2010).

[7] Robledo, L. *et al.* High-fidelity projective read-out of a solid-state spin quantum register. *Nature* **477**, 574–578 (2011).
